# Design, synthesis, *in silico* studies and antiproliferative evaluation of some novel hybrids of pyrimidine-morpholine

**DOI:** 10.3389/fchem.2025.1537261

**Published:** 2025-02-28

**Authors:** Elaheh Ataollahi, Leila Emami, Al-Anood Mohammad Al-Dies, Fateme Zare, Alireza Poustforoosh, Mina Emami, Fateme Saadat, Fateme Motamen, Zahra Rezaei, Soghra Khabnadideh

**Affiliations:** ^1^ Pharmaceutical Sciences Research Center, Shiraz University of Medical Sciences, Shiraz, Iran; ^2^ Department of Chemistry, Al Qunfudah University College, UMM Al-Qura University, Mecca, Saudi Arabia; ^3^ Medicinal and Natural Products Chemistry Research Center, Shiraz University of Medical Sciences, Shiraz, Iran; ^4^ Student Research Committee, School of Pharmacy, Shiraz University of Medical Sciences, Shiraz, Iran; ^5^ Department of Medicinal Chemistry, Faculty of Pharmacy, Shiraz University of Medical Sciences, Shiraz, Iran

**Keywords:** pyrimidine, morpholine, synthesis, simulation, DNA, cytotoxic, cell cycle

## Abstract

**Introduction:**

Cancer is a complex group of diseases characterized by the uncontrolled growth and spread of abnormal cells in the body. These cells can invade nearby tissues and organs, or they may metastasize to other parts of the body through the bloodstream or lymphatic system.

**Methods:**

In this study, eight novel pyrimidine-morpholine hybrides (**2a-2h**) were designed and synthesized based on molecular hybridization approach to identify potent cytotoxic agents. Spectroscopic methods, including infrared spectroscopy (IR), proton and carbon nuclear magnetic resonance (^1^HNMR & ^13^CNMR), and mass spectrometry, were employed to confirm the structures of the compounds. The cytotoxic effects of the derivatives were evaluated against cancerous cell lines, including MCF-7 and SW480, using the MTT assay.

**Results and discussion:**

It was demonstrated that all derivatives had appropriate cytotoxic potential with IC_50_ in range of 5.12–117.04 μM. Compound **2g** was identified as the most potent compound, exhibiting IC_50_ values of 5.10 ± 2.12 μM and 19.60 ± 1.13 μM toward the SW480 and MCF-7 cell lines, respectively. Cell cycle analysis showed that **2g** could induces phase arrest in MCF-7 breast cancer cells. The apoptosis assay demonstrated the induction of apoptosis in the SW480 cell line. The biological activity of the compounds was confirmed by the docking studies. DFT analysis for compounds **2g** and **2h** was conducted at the B3LYP/6-31+G** level of theory. It was concluded that **2g** is both thermodynamically and kinetically more stable than **2h**. Moreover, the interpretation of ADME (Absorption, Distribution, Metabolism, and Excretion) indicates that these new series of compounds possess acceptable prognostic physicochemical properties. These synthesized compounds may serve as promising candidates for further investigation as anticancer agents.

## Introduction

Cancer is a major global health issue that inflicts serious damage on human wellbeing. It is characterized by the unchecked growth of abnormal cells and ranks among the most critical health disorders globally. In fact, cancer has emerged as the second leading cause of death worldwide, with around 9.7 million fatalities reported in 2022 (Gaidai et al., 2023; Ferlay et al., 2021; Siegel et al., 2023). Despite numerous studies conducted globally on cancer treatment, achieving a complete cure remains a significant challenge. Evidence indicates that, even with an understanding of different anticancer products and the effectiveness of these medications, considerable research is yet needed to create targeted drug delivery systems and develop innovative treatments to improve diagnosis and therapy (Debela et al., 2021; Bousbaa, 2021).

**FIGURE 1:**
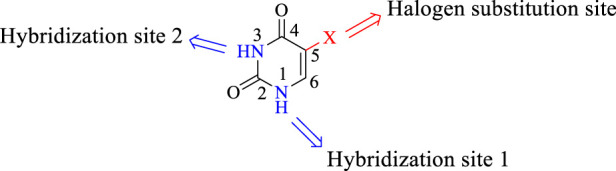
Isatin scaffold and its interaction site.

The strategy of creating a hybrid compound by merging two potent biological molecules into a one has proven to be a very effective way to tackle this serious disease. This approach has garnered the attention of many medicinal chemists (Soltan et al., 2021). Motivated by the outcomes of earlier studies, researchers have recently developed and synthesized pyrimidine hybrids. These hybrids have been tested for their anticancer properties, demonstrating encouraging results against different cancer types (Sanduja et al., 2020). N_1_ and N_3_ are the most common positions in pyrimidine hybrids where other pharmacophores are bound either by a linkage or directly. The C_5_ position, where a halogen can undergo substitution, causes cytotoxic effects (Sanduja et al., 2020; Cardona et al., 2021; Lou et al., 2021). Among the halogen substitutions, fluorine exhibited the greatest inhibitory effect (Rzepiela et al., 2020). Based on literature reports, the cytotoxic effect of 5-FU has been enhanced through the hybridization of different pharmacophores (Moreno-Quintero et al., 2022). In this regard, Figure 1 displays several uracil or 5-FU hybrids of various anticancer drugs (Sanduja et al., 2020; Huang et al., 2012; Jiang et al., 2016; Zhu et al., 2023).

**FIGURE 2:**
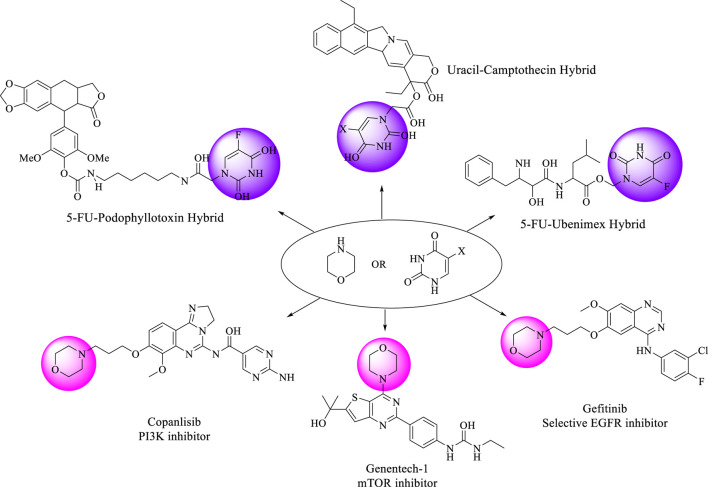
Some drugs and reported candidates with pyrimidine or morpholine scaffold exhibit diverse anticancer effects.

Morpholine (tetrahydro-1,4-oxazine) is recognized as a valuable framework in medicinal chemistry. Due to its diverse biological activities and improved pharmacokinetic and metabolic characteristics, morpholine has emerged as one of the suitable structures being studied in structure-activity relationship (SAR) research (Lou et al., 2021; Kumari and Singh, 2020; Zhang et al., 2022). One of the key advantages of morpholine over other heterocycles with nitrogen atom, is its electrophilic ring, which results from electron-withdrawing effect of oxygen and the relatively weak basicity of nitrogen. Furthermore, morpholine’s capacity to create hydrogen bonds via its oxygen atom is a significant characteristic. The electron-deficient nature of its ring also enables it to engage in hydrophobic interactions (Kourounakis et al., 2020; Tzara et al., 2020). Morpholine and its related compounds have long been recognized for their various activities, such as anticancer, anti-inflammatory, antiviral, antihyperlipidemic, antioxidant, antimicrobial, antipyretic, and analgesic properties (Tzara et al., 2020; Dwivedi et al., 2022; Heiran et al., 2020; Chirra et al., 2024; SHCHERBYNA et al., 2022; Morandini et al., 2021). The antiproliferative activity of various structures containing a morpholine moiety has been extensively studied by researchers (Mao et al., 2017; Kumar et al., 2023; Killock, 2021). Some approved drugs and compounds with anticancer activity containing a morpholine moiety are listed in Figure 2.

Thymidylate synthase (TS) is a crucial enzyme involved in DNA synthesis, playing a significant role in cell survival. TS catalyzes the reductive methylation of dUMP (2′-deoxyuridine monophosphate), resulting in the production of dTMP (deoxythymidine 5′-monophosphate). dTMP undergoes subsequent phosphorylation to form dTTP (deoxythymidine triphosphate), which serves as an intermediate for DNA synthesis. TS is recognized as a target for anticancer agents that contain a pyrimidine group in their structures, used in the treatment of certain cancer cell lines (Song et al., 2021; Ferrari et al., 2018).

In this framework, we designed and synthesized various simple forms of pyrimidine-molpholine hybridizations. Fist different benzyl bromides were conjugated to the hybridization site 1. In the subsequent step to obtain the final compounds, the morpholine ring was joined to the C-6 position of the benzylated pyrimidine ring. Following confirming the structure of the synthesized compounds through spectroscopic methods, we investigated the cytotoxicity of these compounds against two cancer cell lines: MCF-7 (breast carcinoma) and SW480 (colorectal carcinoma) applying MTT assays. Cell cycle analysis and apoptosis assays were conducted to elucidate the mechanism of action. Additionally, eight molecular simulations were conducted to assess the binding energy, interactions and orientations of the compounds with the active site of thymidylate synthase (TS) as a potential therapeutic target. Furthermore, molecular dynamics simulations, density functional theory (DFT) analysis, and the drug-likeness of the compounds were investigated (Jing et al., 2024).

## Experimental section

### Chemistry

All chemical substances were obtained from Merck Company. The melting points of the compounds were determined using the Electrothermal 9200 device (Electrothermal, United Kingdom). The chemical structures of the compounds were analyzed using Infrared spectroscopy (VERTEX70 spectrometer), as well as ^1^HNMR and ^13^CNMR spectra (500 MHz, VARIAN - INOVA Bruker spectrophotometer in CDCl_3_ as solvent).

#### General procedure for the synthesis of 3-methyl-1-(substituted benzyl)-6-morpholinopyrimidine-2,4(1H,3H)-dione derivatives (**2a-2h**)

3-Methyl-6-chlorouracil (1 mmol) was combined with various benzyl bromides (1.2 mmol) (**1a-1h**) and stirred under basic conditions using diisopropylethylamine (DIPEA, 2 mmol) in tetrahydrofuran for 7 h. Upon completion of the reaction, as verified by thin-layer chromatography (TLC), the solvent was removed, yielding the intermediate without further purification (Zhu et al., 2024). Subsequently, the synthesized product from the previous step (1 mmol) was reacted with 1 mmol of morpholine in the presence of a base catalyst (potassium carbonate and triethylamine) in CH_3_CN, and the mixture was refluxed for 24 h. The reaction mixture were then purified through thin layer chromatography (plates) to obtain the purified products. The chemical structures of the synthesized compound (**2a-2h**) were confirmed using ^1^HNMR, ^13^CNMR, and IR spectroscopy.

### Spectra data

#### 1-benzyl-3-methyl-6-morpholinopyrimidine-2,4(1H,3H)-dione (**2a**)

Yield (63%), m.p:149°C–154 °C. yellow powder. ^1^H-NMR (500 MHz, CDCl_3_) δ_H_ (ppm) = 7.33 (d, 2H, *J* = 8 Hz, Phenyl), 7.27-7.29 (m, 1H, Phenyl), 7.22 (d, 2H, *J* = 8 Hz, Phenyl), 5.38 (s, 1H, pyrimidine), 5.12 (s, 2H, CH_2_), 3.75 (t, 4H, *J* = 4 Hz, morpholine), 3.33 (s, 3H, CH_3_-pyrimidine), 2.89 (t, 4H, *J* = 4 Hz, morpholine). ^13^C-NMR (125 MHz, CDCl_3_) δ_C_ (ppm) = 163.28, 159.57, 152.85, 136.74, 128.81, 127.70, 126.69, 125.94, 90.18, 66.11, 51.43, 48.12, 29.70, 28.00. IR (KBr, cm^-1^): 2923.69 (CH aromatic), 2855.02 (CH aliphatic), 1755.53–1692.52 (C=O), 1647.58 (C=N), 1605.02 (C=C), 1453.40 (C-O), 1366.66 (C-N). MS m/z (%):301.3 (57.1), 210.2 (37.5), 91.2 (100), 153.1 (36.3). Elem. Anal. Calcd. For C_16_H_19_N_3_O_3_ (301.35); C, 63.77; H, 6.36; N, 13.94; Found: C, 63.68; H, 6.54; N, 13.87.

#### 1-(3-chlorobenzyl)-3-methyl-6-morpholinopyrimidine-2,4(1H,3H)-dione (**2b**)

Yield (65%), m.p:88°C–90°C. yellow powder. ^1^H-NMR (500 MHz, CDCl_3_) δ_H_ (ppm) = 7.23–7.29 (m, 3H, Phenyl), 7.10–7.12 (m, 1H, Phenyl), 5.39 (s, 1H, pyrimidine), 5.08 (s, 2H, CH_2_), 3.78 (t, 4H, *J* = 4 Hz, morpholine), 3.32 (s, 3H, CH_3_-pyrimidine), 2.89 (t, 4H, *J* = 4 Hz, morpholine). ^13^C-NMR (125 MHz, CDCl_3_) δ_C_ (ppm) = 163.06, 159.26, 152.75, 138.79, 134.71, 130.12, 127.98, 127.02, 124.92, 90.56, 66.09, 51.49, 47.44, 29.71, 28.02. IR (KBr) v (cm^-1^): 2852.69 (CH aromatic), 1706.18 (C=O), 1658.40 (C=N), 1602.96 (C=C), 1441.93 (C-O), 1361.38 (C-N), 765.52 (C-Cl). MS m/z (%): 335.2 (77.0), 249.1 (3.6), 210.1 (76.18), 153.1 (82.9), 125.0 (100), 86.1 (22.3). Elem. Anal. Calcd. For C_16_H_18_ClN_3_O_3_ (335.79); C, 57.23; H, 5.40; N, 12.51; Found: C, 56.97; H, 4.63; N, 12.34.

#### 4-((3-methyl-6-morpholino-2,4-dioxo-3,4-dihydropyrimidin-1(2H)-yl)methyl)benzonitrile (**2c**)

Yield (58%), m.p:202°C–206°C. yellow powder. ^1^H-NMR (500 MHz, CDCl_3_) δ_H_ (ppm) = 7.21–7.24 (m, 2H, Phenyl), 7.02 (t, 2H, *J* = 8 Hz, Phenyl), 5.38 (s, 1H, H_5_-Pyrimidine), 5.07 (s, 2H, CH_2_), 3.78 (t, 4H, *J* = 4 Hz, morpholine), 3.31 (s, 3H, CH_3_), 2.90 (t, 4H, *J* = 4 Hz, morpholine). ^13^C-NMR (125 MHz, CDCl_3_) δ_C_ (ppm) = 163.41, 163.13, 160.96, 159.32, 152.78, 132.48, 128.79, 115.83, 115.62, 90.36, 66.12, 51.47, 47.35, 27.97. IR (KBr, cm^−1^): 2921.41 (CH aromatic), 2854.52 (CH aliphatic), 2227.99 (CN), 1654.52 (C=O), 1448.96 (C-O), 1364.33 (C-N). MS m/z (%): 326.2 (94.5), 240.1 (3.7), 210.1 (87.9), 153.1 (93.5), 116.1 (100), 86.1 (32.3). Elem. Anal. Calcd. For C_17_H_18_N_4_O_3_ (326.36); C, 62.57; H, 5.56; N, 17.17; Found: C, 62.43; H, 5.62; N, 17.32.

#### 3-methyl-1-(4-methylbenzyl)-6-morpholinopyrimidine-2,4(1H,3H)-dione (**2d**)

Yield (53%), m.p:186°C–190°C. yellow powder. ^1^H-NMR (500 MHz, CDCl_3_) δ_H_ (ppm) = 7.10-7.14 (m, 4H, Phenyl), 5.38 (s, 1H, pyrimidine), 5.07 (s, 2H, CH_2_), 3.77 (t, 4H, *J* = 4 Hz, morpholine), 3.32 (s, 3H, CH_3_-pyrimidine), 2.90 (t, 4H, *J* = 4 Hz, morpholine), 2.33 (s, 3H, CH_3_). ^13^C-NMR (125 MHz, CDCl_3_) δ_C_ (ppm) = 163.34, 159.61, 152.83, 137.43, 133.67, 129.46, 126.73, 90.03, 66.14, 51.42, 47.94, 30.19, 29.70, 27.98, 21.11. IR (KBr) v (cm^-1^): 2921.80 (CH aromatic), 2853.04 (CH aliphatic), 1699.15 (C=O), 1654.08 (C=C), 1448.66 (C-O), 1380.51 (C-N). MS m/z (%): 315.1 (70.7), 210.1 (26.5), 153.1 (22.1), 105.2 (100). Elem. Anal. Calcd. For C_17_H_21_N_3_O_3_ (315.37); C, 64.74; H, 6.71; N, 13.32; Found: C, 64.51; H, 6.62; N, 12.97.

#### 1-(4-fluorobenzyl)-3-methyl-6-morpholinopyrimidine-2,4(1H,3H)-dione (**2e**)

Yield (53%), m.p:172°C–175°C. yellow powder. ^1^H-NMR (500 MHz, CDCl_3_) δ_H_ (ppm) = 7.46 (d, 2H, *J* = 8 Hz, Phenyl), 7.11 (d, 2H, *J* = 8 Hz, Phenyl), 5.38 (s, 1H, H_5_-Pyrimidine), 5.05 (s, 2H, CH_2_), 3.76 (t, 4H, *J* = 4 Hz, morpholine), 3.31 (s, 3H, CH_3_), 2.89 (t, 4H, *J* = 4 Hz, morpholine). ^13^C-NMR (125 MHz, CDCl_3_) δ_C_ (ppm) = 163.06, 159.26, 152.76, 135.75, 131.94, 128.56, 121.66, 90.38, 66.10, 51.45, 47.47, 27.99. IR (KBr, cm^−1^): 2967.41 (CH aromatic), 2918.82–1857.15 (CH aliphatic), 1699.62 (C=O), 1654.51 (C=N), 1507.24 (C=C), 1448.68 (C-O), 1361.68 (C-N), 1160.54(C-F). MS m/z (%): 319.1 (3.5), 227.1 (100), 199.1 (88.5), 109.1 (33.6). Elem. Anal. Calcd. For C_16_H_18_FN_3_O_3_ (319.34); C, 60.18; H, 5.68; N, 13.16; Found: C, 60.32; H, 5.83; N, 13.37.

#### 1-(4-bromobenzyl)-3-methyl-6-morpholinopyrimidine-2,4(1H,3H)-dione (**2f**)

Yield (78%), m.p: °C. yellow powder. ^1^H-NMR (500 MHz, CDCl_3_) δ_H_ (ppm) = 7.62 (d, 2H, *J* = 8 Hz, Phenyl), 7.33 (d, 2H, *J* = 8 Hz, Phenyl), 5.40 (s, 1H, H_5_-Pyrimidine), 5.14 (s, 2H, CH_2_), 3.74 (t, 4H, *J* = 4 Hz, morpholine), 3.30 (s, 3H, CH_3_), 2.86 (t, 4H, *J* = 4 Hz, morpholine). ^13^C-NMR (125 MHz, CDCl_3_) δ_C_ (ppm) = 162.90, 159.04, 152.65, 142.15, 127.38, 118.40, 111.70, 90.61, 66.04, 51.45, 47.60, 28.01. IR (KBr, cm^-1^): 3095.17 (CH aromatic), 2960.05–2852.97 (CH aliphatic), 1696.07 (C=O), 1654.36 (C=N), 1448.17 (C-O), 1358.80 (C-N), 723.91 (C-Br). MS m/z (%): 381.1 (56.0), 210.1 (70.7), 171.0 (100), 153.1 (44.8),. Elem. Anal. Calcd. For C_16_H_18_BrN_3_O_3_ (380.24); C, 50.54; H, 4.77; N, 11.05; Found: C, 50.37; H, 4.82; N, 11.35.

#### 3-methyl-6-morpholino-1-(4-(trifluoromethyl)benzyl)pyrimidine-2,4(1H,3H)-dione (**2g**)

Yield (75%), m.p:144°C–150°C. white powder. ^1^H-NMR (500 MHz, CDCl_3_) δ_H_ (ppm) = 7.60 (d, 2H, *J* = 8 Hz, Phenyl), 7.34 (d, 2H, *J* = 8 Hz, Phenyl), 5.40 (s, 1H, pyrimidine), 5.16 (s, 2H, CH_2_), 3.76 (t, 4H, *J* = 4 Hz, morpholine), 3.32 (s, 3H, CH_3_-pyrimidine), 2.90 (t, 4H, *J* = 4 Hz, morpholine). ^13^C-NMR (125 MHz, CDCl_3_) δ_C_ (ppm) = 162.97, 159.18, 152.72, 140.79, 130.16, 129.84, 127.04, 125.79, 125.27, 122.57, 90.48, 66.07, 51.46, 47.55, 27.99. IR (KBr) v (cm^−1^): 2981.94 (CH aromatic), 2857.32 (CH aliphatic), 1656.56 (C=O), 1452.00 (C-O), 1370.02 (C-N), 1114.99 (C-F). MS m/z (%): 369.2 (72.3), 210.1 (80.3), 153.1 (100), 159.2 (98.8). Elem. Anal. Calcd. For C_17_H_18_F_3_N_3_O_3_ (369.34); C, 55.28; H, 4.91; N, 11.38; Found: C, 55.41; H, 5.12; N, 11.56.

#### 3-methyl-6-morpholino-1-(4-nitrobenzyl)pyrimidine-2,4(1H,3H)-dione (**2h**)

Yield (60%), m.p:141°C–146°C. yellow powder. ^1^H-NMR (500 MHz, CDCl_3_) δ_H_ (ppm) = 8.16 (s, 2H, Phenyl), 7.61 (d, 1H, *J* = 8 Hz, Phenyl), 7.54 (t, 1H, *J* = 8 Hz, Phenyl), 5.42 (s, 1H, pyrimidine), 5.18 (s, 2H, CH_2_), 3.81 (t, 4H, *J* = 4 Hz, morpholine), 3.30 (s, 3H, CH_3_-pyrimidine), 2.92-2,95 (m, 4H, morpholine). ^13^C-NMR (125 MHz, CDCl_3_) δ_C_ (ppm) = 162.83, 158.92, 152.62, 148.42, 138.77, 133.28, 129.92, 122.91, 122.22, 90.95, 66.08, 51.58, 47.04, 29.69, 28.00. IR (KBr) v (cm^−1^): 2963.56 (CH aromatic), 2854.22 (CH aliphatic), 1658.11 (C=O), 1441.38 (C-O), 1527.77 (O=N=O), 1350.38 (C-N). MS m/z (%): 346.1 (99.2), 210.1 (100), 153.1 (95.8), 136.1 (68.1). Elem. Anal. Calcd. For C_16_H_18_N_4_O_5_ (346.34); C, 55.49; H, 5.24; N, 16.18; Found: C, 55.37; H, 5.18; N, 16.34.

### Biological evaluations

#### MTT assay

The MTT assay, which stands for 3-(4,5-Dimethylthiazol-yl)-2,5-Diphenyl-Tetrazolium Bromide, was employed to assess the antiproliferative effects of the synthesized derivatives (**2a-2h**) based on our established methods (Ferlay et al., 2021; Zhu et al., 2024). The cancerous cell lines MCF-7 and SW480 were sourced from the National Cell Bank of Iran (NCBI). Cancer cell lines were cultured in RPMI 1640 media supplemented with 10% fetal bovine serum (FBS) and 1% penicillin-streptomycin (Gibco, USA). Cells were harvested using a 0.5% trypsin/EDTA solution (Gibco, USA). The cells were then plated at a density of 1 × 10^4^ cells per well in 96-well microplates (Zare et al., 2023). Treatment involved five different concentrations of the derivatives and cisplatin (ranging from 1 to 200 μM), with each concentration tested in triplicate. Three wells were left untreated to serve as negative controls. Later than 48 h, the media was replaced with 100 μL of a freshly prepared MTT mixture and incubated for 4 h at 37°C to facilitate for the formation of purple formazan crystals (Ataollahi et al., 2024). The medium was then discarded, and 150 μL of dimethyl sulfoxide was added to dissolve the crystals, followed by a 10-minute incubation at 37°C in the dark. Absorbance was measured at 490 nm using a microplate ELISA reader. Data analysis was performed using Excel 2016 and Curve Expert 1.4, with results presented as mean ± SD for each analysis (Poustforoosh, 2025).

#### Apoptosis

A total of 5 × 10^4^ SW480 cells were precultured in 24-well plates in RPMI 1640 culture medium for 16 h under standard culturing conditions. After treating cells with 2.5, 5.0, and 10.0 µM of the **2g** complex, the cells were harvested using 0.25% Trypsin, washed with PBS, and stained with propidium iodide (5 μL, 1 mg/mL), and 1U of RNase A. Cell cycle analysis was performed using flow cytometry (BD FACSCalibur Flow Cytometer, BD, USA). The DNA content was analyzed using FlowJo 10.0 software (Zhao et al., 2024a).

#### Cell cycle

The total number of 5 × 10^4^ SW480 cells were precultured in 24-well plates in RPMI 1640 culture medium for 16 h under standard culturing conditions. After treating cells with 2.5, 5.0, and 10.0 µM of the **2g** complex, the cells were harvested using 0.25% Trypsin, washed with PBS, and fixed in 70% ethanol for 2 h. The cells were then centrifuged at 4,000 rpm for 2 min. In the next step, cells were permeabilized with 0.2% Triton X-100 for 15 min at 4°C. After centrifugation at 4,000 rpm for 2 min, the cells were resuspended in PBS containing propidium iodide (5 μL, 1 mg/mL) and 1U of RNase A. Cell cycle analysis was performed using flow cytometry (BD FACSCalibur Flow Cytometer, BD, USA). The DNA content was analyzed using FlowJo 10.0 software.

### Computational studies

#### Molecular docking

The crystal structure of Thymidylate synthase (TS) was obtained from the RCSB Protein Data Bank (PDB ID: 1HVY) (Phan et al., 2001). The molecular docking process was carried out using AutoDock Vina. The compounds’ structures were energy-minimized and converted to pdbqt format. For the docking analysis, a grid box measuring 40 × 40 × 40 Å and an exhaustiveness setting of 100 were applied. Discovery Studio 2016 was employed to visualize the interactions and orientations of the compounds.

#### MD studies

The interactions between **2g**, **2h**, and 5-FU and human thymidylate synthase (PDB ID: 1HVY) were further evaluated in a dynamic context. **2g** was the most active compound, **2h** was the compound with the lowest activity, and 5-FU was used as the positive control. Desmond by Schrödinger was used for molecular dynamics (MD) simulation. The complex system obtained from the docking analysis was utilized for the molecular dynamics simulation. The simulation was conducted within an orthorhombic box, and the solvent model used for the simulation was the Transferable Intermolecular Potential with 3 Points (TIP3P). By employing Schrödinger’s system setup, the system was successfully neutralized using the precise amount of Na^+^/Cl^−^ ions, which had a salt concentration of 0.15 M (Zhao et al., 2024a). Following the default relaxation protocol of the software, the simulation was carried out for a total of 100 nanoseconds, with the number of atoms, pressure, and temperature held constant in the NPT ensemble. Utilizing the Nose-Hoover thermostat, the temperature was precisely set to 310.15 K (37°C), and the pressure was meticulously adjusted to 1 atm through the implementation of isotropic scaling.

#### DFT study

Gaussian 09 was employed at the B3LYP/6-31+G** theoretical level to optimize the geometries of **2g** and **2h** without considering symmetry. DFT analyses provided calculations for total energy (Etot), enthalpy (H°), Gibbs free energy (G°), entropy (S), softness (σ), hardness (η), and electron affinity (A) at the B3LYP/6-31+G** level and a temperature of 298K.

#### Pharmacokinetic profiles

The physicochemical characteristics of all the synthesized compounds were assessed using the online platform http://www.swissadme.ch/, following the guidelines of Lipinski and Veber.

## Results and discussion

### Chemistry

**FIGURE 3:**
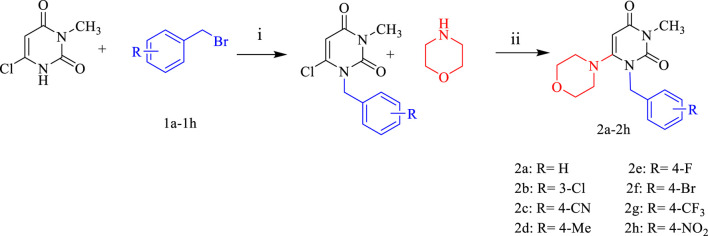
The standard reactants and condition for the synthesis of derivatives **2a-2h** are as follows: i: DIPEA in THF for 6 h at 40°C, ii: K_2_CO_3_/Et_3_N in CH_3_CN for 24 h under reflux.

Eight new pyrimidine-morpholine hybrid compounds were synthesized through a two-step process. First, various benzyl bromides (**1a-1h**) were reacted with 3-methyl-6-chlorouracil in tetrahydrofuran (THF) at 25°C to produce the benzylated pyrimidine intermediates. The ultimate derivatives (**2a-2h**) were obtained by reacting the benzylated pyrimidine with morpholine in a basic reaction system that included triethylamine and potassium carbonate, under reflux conditions for an overnight period. Figure 3 depicts the representation of the synthetic process for these compounds.

In ^1^H-NMR spectra, the significant piece of compounds **2a-2h** are a single peak belong to the proton of pyrimidine at 5.07–5.18 ppm. Two protons of CH_2_ was observed as singlet at 5.38–5.42 ppm. A key characteristic of the 13C NMR spectrum for these compounds is associated with C5 of the pyrimidine ring, which is observed in the range of 90.04–90.95 ppm.

### Biological activity

#### MTT assay

**FIGURE 4:**
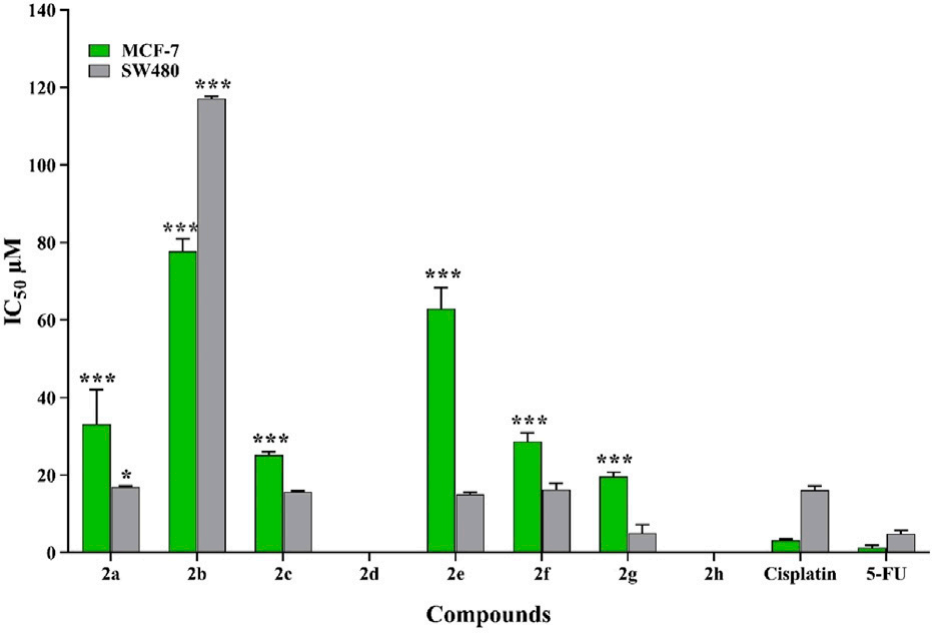
The toxic impact of the created pyrimidine-morpholine hybrids on MCF-7 and SW480 cell lines was evaluated in relation to Cisplatin and 5-FU. (*:p value <0.05, **:p value <0.01, ***:p value <0.001).

The antiproliferative activity of eight novel pyrimidine-morpholine derivatives (**2a-2h**) were evaluated toward two human cancerous cell lines, SW480 and MCF-7. The results can be found in Figure 4 and Table 1. 5-Fluorouracil (5-FU) and Cisplatin were used as positive controls. The most potent compound was (**2g**), which demonstrated an IC_50_ value of 5.10 ± 2.12 μM, compared to 5-FU (IC_50_ = 4.90 ± 0.83 μM) and Cisplatin (IC_50_ = 16.10 ± 1.10 μM) in the SW480 cell line. Additionally, compounds **2c** and **2e** exhibited significant cytotoxic potential compared to Cisplatin. These pyrimidine-morpholine derivatives displayed higher cytotoxic activity in the SW480 cell line than in the MCF-7 cell line.

**TABLE 1:** Antiproliferative properties of the pyrimidine-morpholine compounds (**2a-2h**).

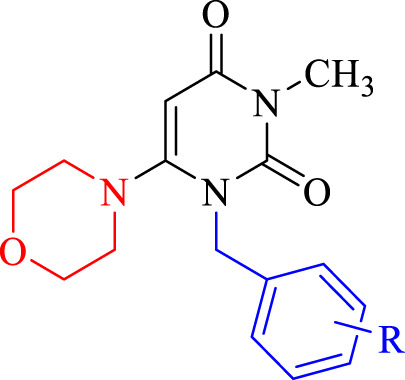			
Entry	R	IC_50_ ± SD (µM)	
		MCF-7	SW480
**2a**	H	33.15 ± 8.90	16.90 ± 0.28
**2b**	3-Cl	77.75 ± 3.18	117.05 ± 0.64
**2c**	4-CN	25.21 ± 0.85	15.72 ± 0.28
**2d**	4-Me	>200	>200
**2e**	4-F	62.90 ± 5.51	15.05 ± 0.49
**2f**	4-Br	28.65 ± 2.19	16.31 ± 1.56
**2g**	4-CF_3_	19.62 ± 1.13	5.10 ± 2.12
**2h**	4-NO_2_	>200	>200
Cisplatin	—	3.21 ± 0.27	16.10 ± 1.10
**5-FU**	—	1.30 ± 0.56	4.90 ± 0.83

Structure-activity relationship explained that compound **2g** with CF_3_ substituation (electron-withdrawing group) at *para* position of the phenyl ring showed the best cytotoxic effect. Fluorine, bromine and nitrile, as electronegative substitutions on *para* position of the phenyl ring, enhanced the toxic effect, too. The presence the eletrondonating groups in compound (**2d**) led to deteriorate the activity compared to unsubstituted derivative (**2a**). When the 4-NO_2_ group was introduced on the benzyl ring, a notable decline in the activity was observed, which may be due to the large size of this functional group. The existance of eletronnegative group at *meta* position resulted in compound **2b**, cause to decrease activity. Taken togather, it was understood that the existence of electron donating substitutions at *para* position and movement the substituation from *para* to *meta* position falling down the potency and the electronnegative groups at the *para* position empowered the cytotoxic activity. These results highlight the significance of electron-withdrawing substitutions in pyrimidine-morpholine compounds for their anticancer activities. Conversely, electron-donating substitutions and substitutions with large sizes were found to decrease the activity.

#### Apoptosis

**FIGURE 5:**
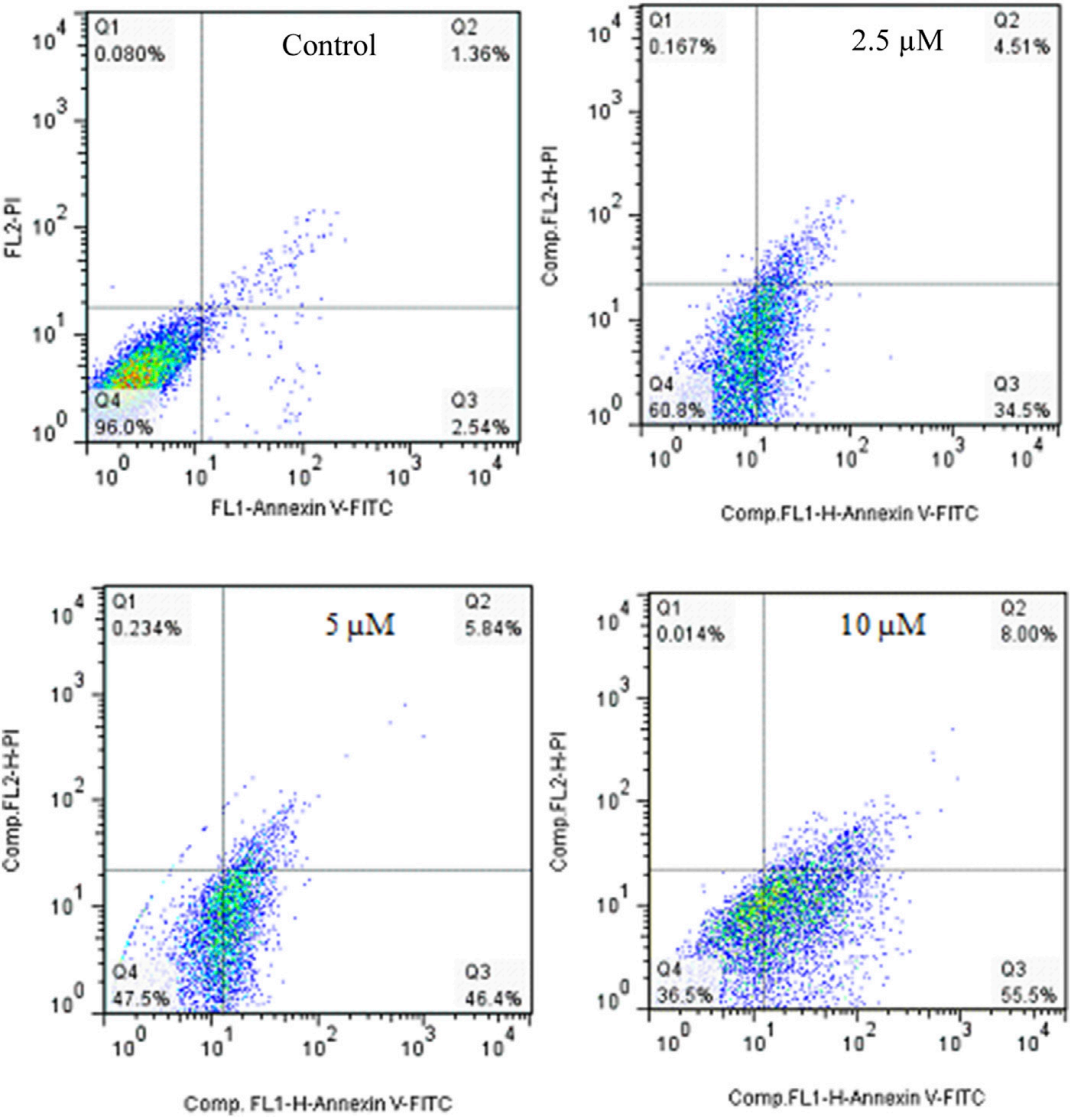
Flow cytometric studies of the apoptotic effects of 2g on the SW480 cell line after 72 h of treatment.

Annexin V-Propodium Iodide (PI) double staining technique was used to determine the cell death mechanism. This technique diagnosis four staining pattern: (Av^neg^/PI^neg^):viable:, (Av^pos^/PI^neg^ early apoptotic, (Av^pos^/PI^pos^): late apoptotic and (Av^neg^/PI^pos^): necrotic cells. In apoptosis stage, membrane inversion phenomena could cause that phosphatidylserine was moved to the outer membrane and so, deteted with AV-FITC staining. PI was utilized to detect both live and dead cells. The apoptotic potential of compound **2g** on SW480 cells was assessed at three different concentrations (2.5, 5, and 10 µM) following a 72-hour treatment period. The results indicated that **2g** significantly increased the percentage of Annexin V-positive cells from 3.9% to 39.1%, 52.24%, and 63.5%, demonstrating its ability to induce apoptosis in the SW480 cell line in a concentration-dependent modes (Figure 5).

**FIGURE 6:**
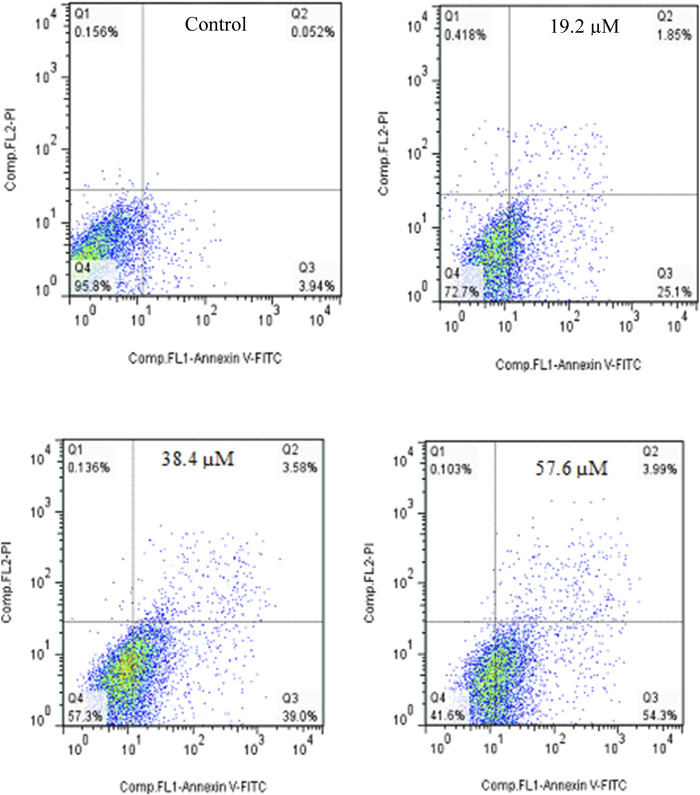
Flow cytometric studies of the apoptotic effects of Cisplatin on the SW480 cell line after 72 h of treatment.

The apoptosis effect of Cisplatin as positive control was performed and is shown in Figure 6. As could be seen, Cisplatin induce apoptosis and increased the percentage of Annexin V-positive cells from 3.99% to 27.35%, 42.58%, and 58.29%. taken together, **2g** could induce apoptosis in lower concetration compared to Cisplatin.

### Cell cycle assessment

**FIGURE 7:**
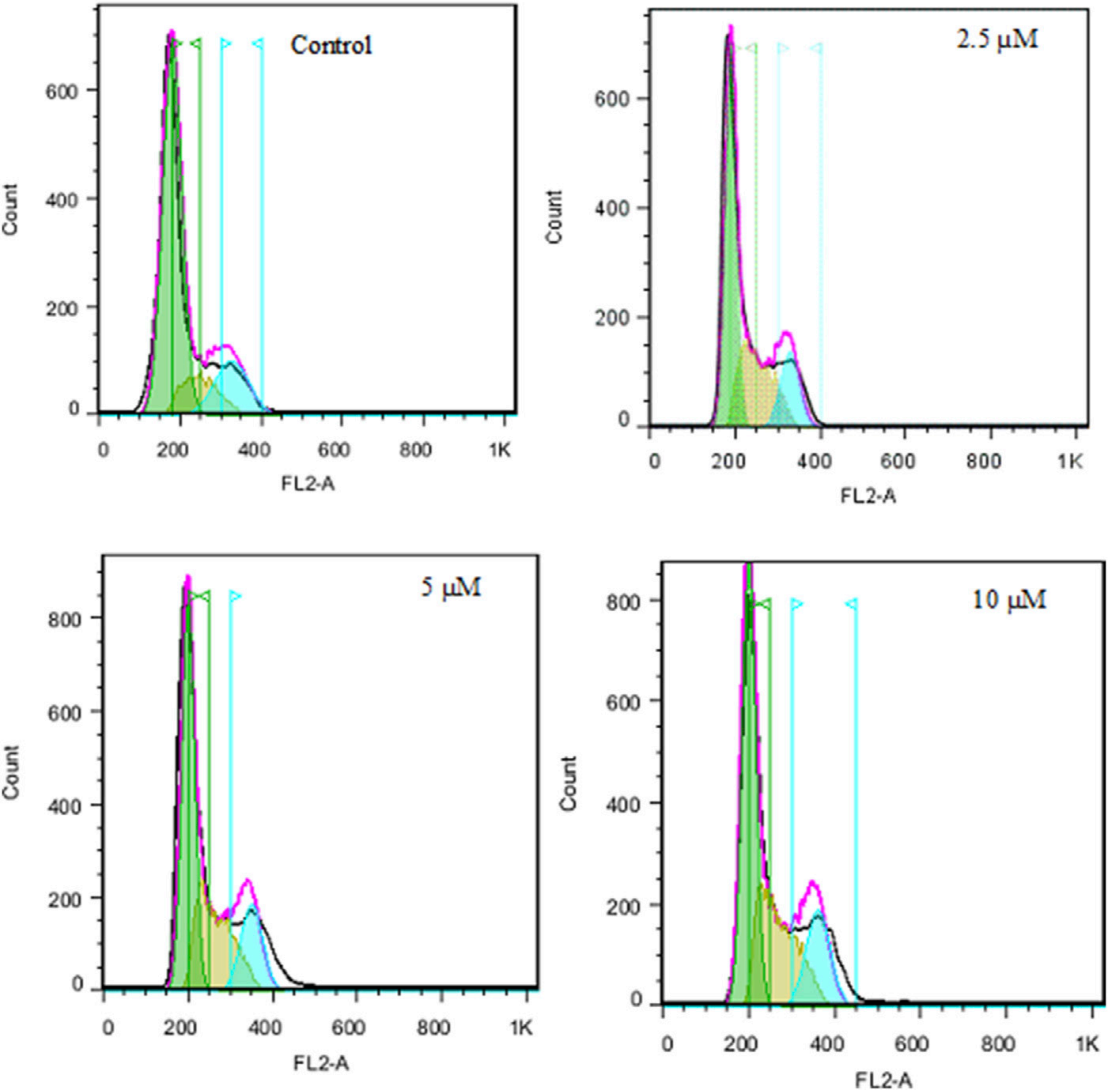
The effect of **2g** on cell cycle in SW480 cell line after 72 h treatment.

The cell cycle approach was employed to examine how cells are distributed among various phases (G0, G1, S, G2, M) through flow cytometry. As shown in Figure 7, SW480 cells that were treated with compound **2g** showed a rise in the population of cells in the S-phase, with percentages of 24.41%, 29.34%, and 34.38% after treatment with 2.5, 5 and 10 μM, respectively, compared to untreated cells, which had a percentage of 15.50%. These findings indicate that compound **2g** can induce an arrest in the S-phase (Table 2).

**TABLE 2:** The impact of **2g** on the cell cycle of the SW480 cell line following a 72-hour treatment period.

Sample		SubG1/G1	S	G2/M	
1	Control	71.94	15.50	12.56	-
4	2.5 µM	58.73	24.41	16.86	Cell cycle arrest at the S phase
3	5 µM	55.08	29.34	15.58	
5	10 µM	48.88	34.38	16.74	

### Computational studies

#### Molecular docking study

**FIGURE 8:**
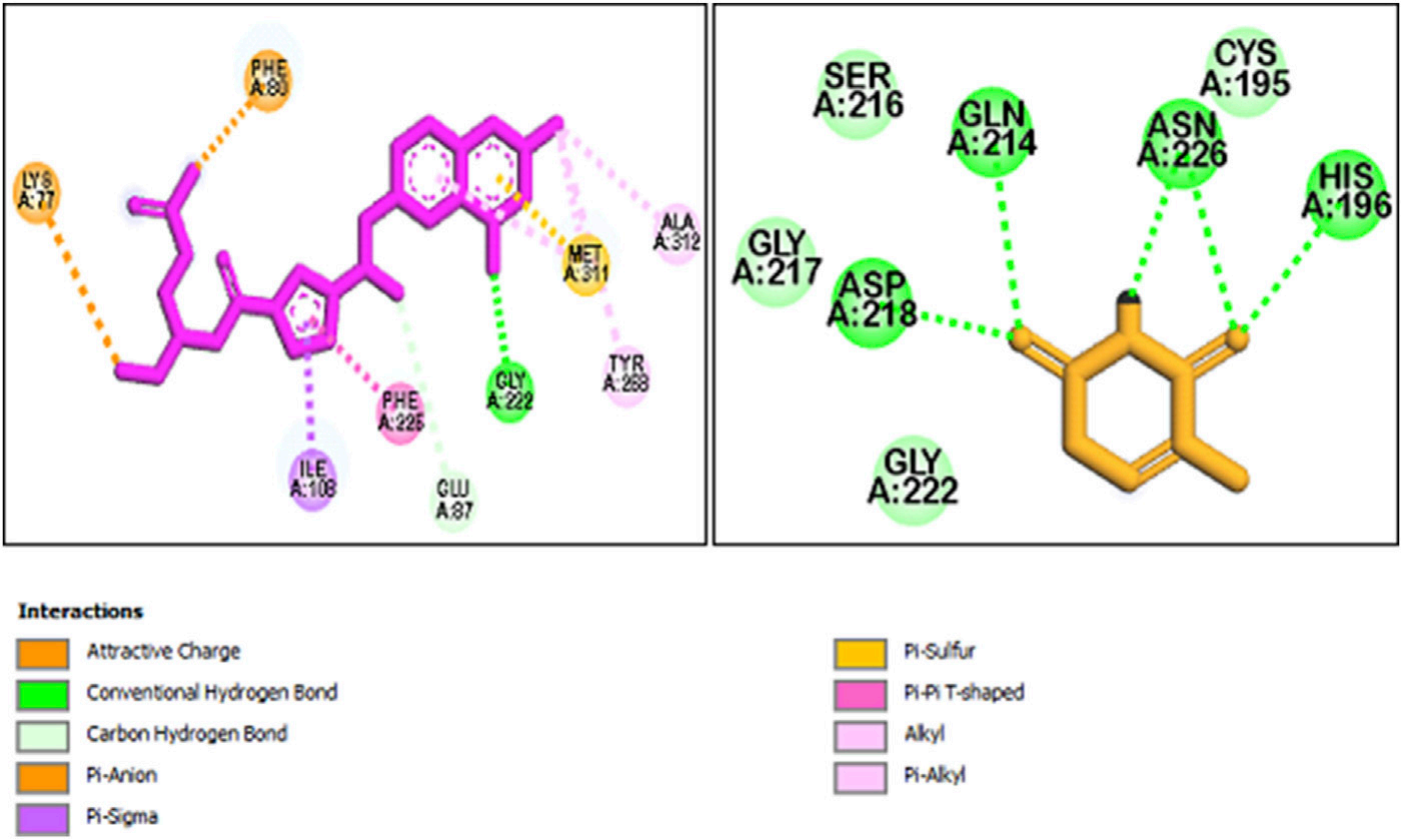
The interaction and orientation of internal ligand (D16) and 5-FU in the active site of 1hvy.

Molecular docking research offers a deeper insight into how molecules behave, their binding configurations, and their interactions with receptors. This leads to a more thorough analysis of the biological behavior of the compounds under study (Emami et al., 2024). All synthetic compounds were docked onto the human thymidylate synthase complexed with Raltitrexed (D16) with PDB ID: 1hvy. To determine the orientation of the synthesized compounds and the important amino acids involved in receptor binding, the interactions and positioning of the receptor’s internal ligand (D16) and 5-FU were initially examined. The results are presented in Figure 8.

As observed, the compound D16 interacted through hydrogen bonding and π interactions with key residues, namely Lys 77, Phe 80, Met 311, Gly 222, Phe 226, and Ile 108. Also, for a better interpretation of the docking results of the studied compounds, docking studies were also performed on the current anticancer drug 5-FU. That is located in the binding pocket, interacting with residues such as Ser 216, Gly 217, Gly 222, and Cys 195 through hydrophobic interactions. Additionally, it forms critical hydrogen bonds with residues Asn 226, His 196, Gln 214, and Asp 218.

**FIGURE 9:**
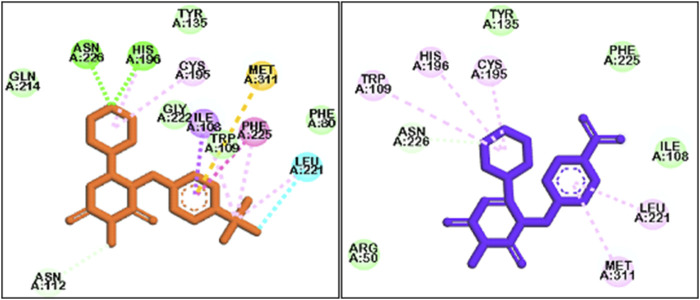
The interaction and orientation of **2g** (Red) and **2h** (Blue) in the active site of 1hvy.

The interactions and the placement of the most and least active compounds **2g** and **2h** with 1hvy are illustrated in Figure 9. Furthermore, the docking results of all compounds, including the binding energies, hydrogen bonding, and hydrophobic and π interactions, are presented in Supplementary Tables S1, S2, respectively. As expected, compound **2g** has established strong interactions with the receptor and has interacted with most of the key amino acids. The binding energy for **2g** was determined to be −8.6 kcal/mol, significantly higher than the value obtained for 5-FU (−5.2 kcal/mol). In the 2g, the morpholine ring connected to pyrimidine is situated in the binding pocket similarly to compound 5-FU. It interacts with the key amino acids His 196 and Asn 226 through hydrogen bonding. Moreover, it can be seen that most of the interactions of compound **2g** with the receptor occur in the region where the trifluorophenyl substitution is located. This substitution interacts with the amino acids Cys 195, Phe 225, Ile 108, Met 311, and Leu 213 through π interactions.

The binding energy for compound **2h** was determined to be −7.6 kcal/mol, which is the lowest value of the binding energy among all the compounds studied. Figure 9 illustrates that this compound is located in the binding pocket but establishes weak interactions with the receptor 1hvy. Important amino acids Asn 226 and His 196 are positioned around the morpholine ring connected to pyrimidine, but this motif was unable to form strong hydrogen bonds as observed in compound **2g**. Also, the nitrophenyl substitution, which likely played a crucial role in orienting this compound, did not result in the anticipated key interactions. Overall, the docking results are in good agreement with the biological findings.

### MD simulation

**FIGURE 10:**
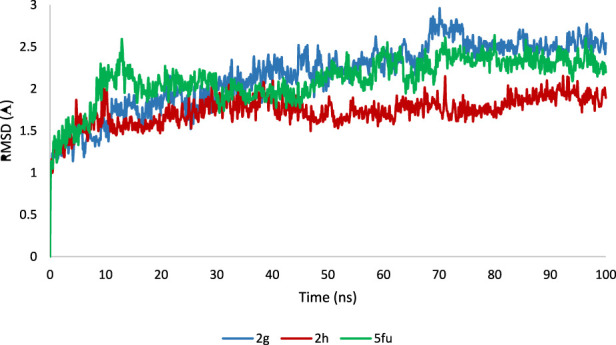
The Root Mean Square Deviation (RMSD) values of the enzyme during a 100 ns Molecular Dynamics (MD) simulation for the ligand-enzyme complexes of **2g** (blue), **2h** (red), and 5FU (green).

The ligand-enzyme complex involving three compounds (2g, 2h, and 5-FU) underwent additional assessment through a 100-nanosecond MD simulation (Poustforoosh, 2024). Figure 10 displays the RMSD value for the 2g-enzyme complex during the simulation. The RMSD values show variations within a small range of 1.5–2.5 Å, suggesting that the system remained stable throughout the simulation duration.This value is almost the same for 5-fluoropyrimidine. For **2h**, similar to **2g** and 5-FU, the RMSD values fluctuate around 1.5–2 Å, but their variations are smoother compared to **2g** and 5-FU. These values indicate that simulations have reached equilibrium after some time (Kang et al., 2018; Duan et al., 2024; Gao et al., 2023).

**FIGURE 11:**
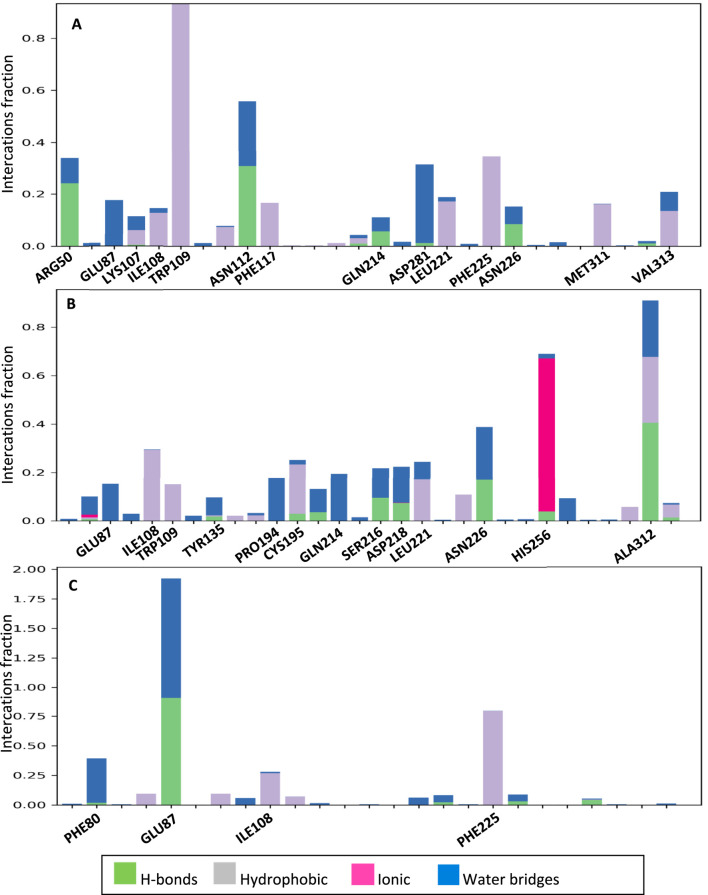
The interactions formed between the ligands and proteins during a 100 ns molecular dynamics (MD) simulation for **2g (A)**, **2h (B)**, and 5FU **(C)**.

Figure 11A shows the interactions that occurred between **2g** and the protein over a duration of 100 ns. The residues that exhibited the most significant interaction fraction are Trp109 and Asn112.Trp 109 is a crucial residue in the active site of thymidylate synthase, regulating its catalytic activity. Therefore, targeting this residue is an effective strategy for enzyme inhibition. Moreover, Tong et al. have shown that Trp109 and Asn112 are two essential residues, and targeting them can be used for enzyme inhibitors. In the case of compound **2h**, the residues exhibiting the highest interaction fraction are Ala312 and His256 (refer to Figure 11B). This observation may elucidate the diminished inhibitory activity of this compound in comparison to **2g**. The main target residues for 5-fluoropyrimidine are Glu87 and Phe225 (Figure 11C). Glu87 is also a crucial residue for targeting and enzyme inhibition.

**FIGURE 12:**
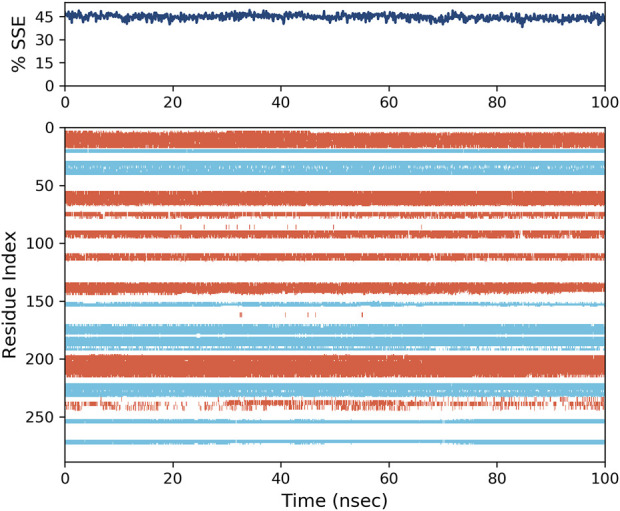
The variation in the percentage of secondary structure elements (SSE) for the protein throughout the molecular dynamics (MD) simulation is presented. The regions depicted in red correspond to alpha-helices, while those in blue indicate beta-strands.

Another important parameter that needs to be evaluated for the simulation is the percentage of secondary structure elements (SSE), which serves as an indicator of the structural compactness (Tong et al., 2022; Yin et al., 2025; Zhu et al., 2024). This parameter can be utilized to evaluate the stability of the protein structure throughout the simulation process (Zhang et al., 2018; Zhao et al., 2024b). The activity of the **2g** -protein complex was evaluated, and it was found to be the most active compound. The protein’s alpha-helix structures, indicated by the red zone in Figure 12, and beta strands (colored in blue) exhibited minimal alterations during the simulation. As illustrated in this Figure, the proportion of SSE in the simulation is approximately 45%, a value that has remained consistent throughout the duration of the simulation. This constancy suggests a stable enzyme structure (Poustforoosh, 2025; Li et al., 2024).

### DFT analysis

**FIGURE 13:**
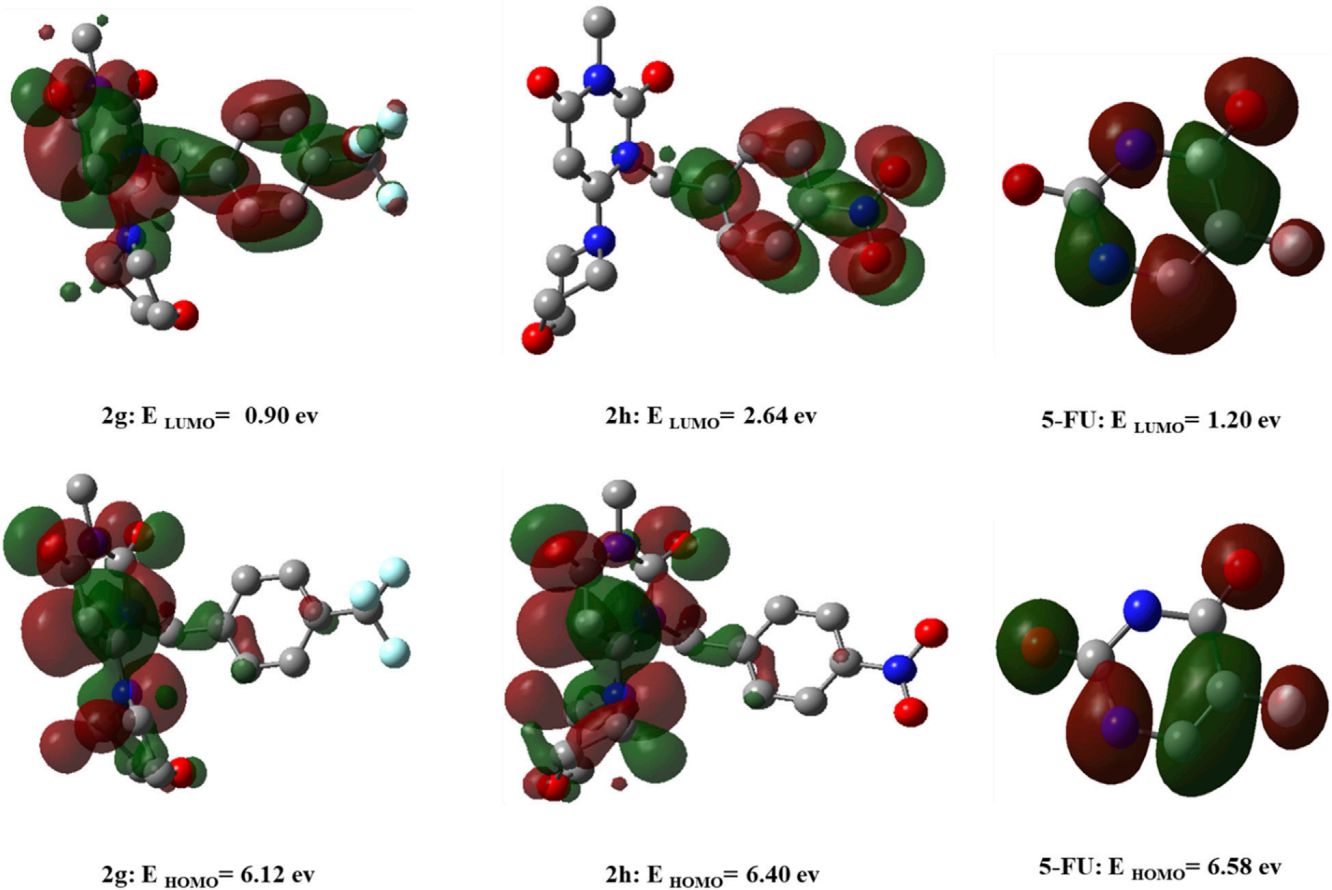
DFT calculated LUMO, HOMO, and their corresponding energy values for compounds **2g**, **2h**, and **5-FU** utilizing the B3LYP/6-31+G (d,p) computational method.

The HOMO and LUMO orbitals for **2g**, **2h**, and **5-FU** are depicted in Figure 13. In **2g**, HOMO is delocalized on morpholine, pyrimidine, and part of triflourophenyl ring, while LUMO is delocalized over the structure of the molecule. For compound 2h, the HOMO orbital is distributed over the morpholine and pyrimidine rings, similar to compound **2g**, while the LUMO orbital is distributed over the nitrophenyl group. For 5-FU, HOMO and LUMO orbitals are delocalized throughout the molecule. The energies of HOMO, LUMO orbitals for **2g**, **2h**, and 5-FU are presented in Figure 13. Thus, the energy gap is calculated as 5.221 eV, 3.753 eV, and 5.387 eV for **2g**, **2h**, and 5-FU, respectively. The energy difference between the HOMO and the LUMO serves as an indicator of the kinetic stability and reactivity of the compounds. In this study, the energy gap order is 5-FU > **2g** > **2h**, indicating that 5-FU is more kinetically stable than **2g**, and consequently, **2g** is more stable than **2h**.

**FIGURE 14:**
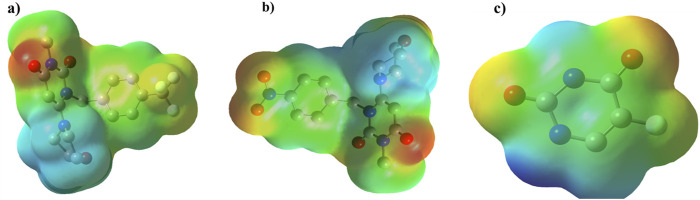
The geometry optimization and electrostatic potential (ESP) calculations for compounds **(A)** 2g, **(B)** 2h, and **(C)** 5-fluorouracil (5-FU) using the B3LYP/6-31+G (d,p) level of theory.

ESP plot for **2g**, **2h**, and 5-FU is indicated in Figure 14, where regions with higher electronegativity are displayed in red, and regions with lower electronegativity are displayed in blue.

Through DFT calculations, valuable insights into structural stability and reactivity can be acquired. By utilizing HOMO and LUMO energies, one can calculate parameters such as hardness (η), softness (s), and electron affinity (A). These parameters provide insights into the interplay among energy, structure, and reactivity characteristics. These parameters, along with total energy (E tot), Gibbs energy (G°), enthalpy (H°), and entropy (S), are provided in Table 3. According to the table, compound **2g** is thermodynamically more stable than **2h** and 5-FU (**2g** > **2h** > 5-FU). A hard molecule has a greater energy gap between HOMO and LUMO and is more resistant to changes in the shape of the electron cloud during chemical reactions. In contrast, a soft molecule has a lower energy gap and is more reactive. Therefore, in this study, the order of hardness of compounds is as follows. 5-FU > **2g** > **2h**. Electron affinity is obtained by using the energy value of LUMO. A reduced electron affinity signifies a diminished propensity to attract electrons, which consequently enhances the likelihood of engaging in nucleophilic reactions. The findings indicate that compound **2g** exhibits a greater inclination to partake in nucleophilic reactions in comparison to the other two compounds.

**TABLE 3:** The calculated total energy (E_tot_), Enthalpy (H), Gibbs free energy (G), hardness (*η*), softness (σ, and electron affinity (A) of **2a**, **2g**, and **5-FU** at B3LYP/6-31+G(d,p) level of theory.

Entry	E_tot_ ^a^	H^a^	G^a^	S^b^	*η* ^c^	σ^d^	A^c^
**2g**	−1340.379	−1340.378	−1340.456	163.680	2.61	0.38	0.899
**2h**	−1208.506	−1208.505	−1208.581	160.859	1.87	0.53	2.647
**5-FU**	−511.124	−511.123	−511.162	82.542	2.70	0.37	1.202

^a^ In Hartree/particle.

^b^ In cal/mol.K.

^c^ In ev.

^d^ In ev−1.

**FIGURE 15:**
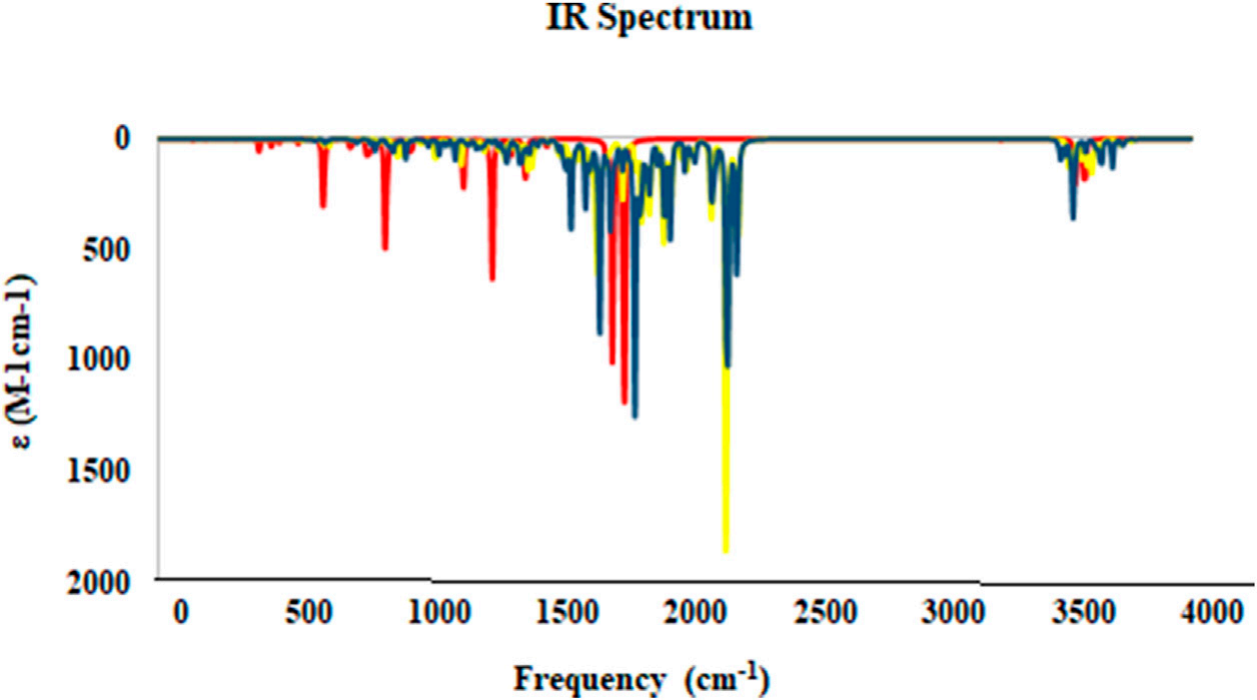
The IR Spectrum for studied compounds **2g** (blue), **2h** (yellow), and **5-FU** (red) was calculated at at B3LYP/6-31+G (d,p) level of theory.

The IR spectra of molecules were obtained through theoretical calculations. The frequencies were determined utilizing the B3LYP/6-311++G (d,p) theoretical framework, as illustrated in Figure 15. The assignment of bands showed C=O stretching vibrations at 1658.11 cm^−1^ (IR), 1696 cm^−1^ (DFT) for **2h**, and 1656.56 cm^−1^ (IR), 1648 cm^−1^ (DFT) for **2g**. The band of O=N=O appeared at 1527.77 cm^−1^ (IR) and 1560 cm^−1^ (DFT) for **2h** and bands of C-F appeared at 1114.99 cm^−1^ (IR) and 1088 cm^−1^ (DFT) for compound **2g**. The stretching modes of C-H aromatic, C-H aliphatic, C-O, and C-N groups have been observed to closely match the frequencies in experimental IR spectra.

### Pharmacokinetic profiles

In the realm of drug design and discovery, computational methodologies are employed to forecast various properties, including molecular weight, LogP, the count of hydrogen bond acceptors, the count of hydrogen bond donors, the number of rotatable bonds, and the topological polar surface area (TPSA). This measure is implemented to avoid the design and synthesis of compounds that do not adhere to Lipinski’s Rule of Five. PreADMET software and the SwissADME online server are valuable tools for predicting the pharmacokinetics and toxicity of designed compounds. These tools can assist researchers in developing drugs that are well-absorbed orally. In this study, the molecular weight of all synthesized compounds was less than 500, the number of hydrogen bond donors (HBD), hydrogen bond acceptors (HBA), and rotatable bonds (n-RB) were all less than 10, and TPSA was less than 140. All compounds meet the Lipinski Rule of Five parameters (Table 4).

**TABLE 4:** Physicochemical properties of the pyrimidine-morpholine derivatives (**2a-2h**).

Entry	MW	MLogP	HBD	HBA	n-RB	TPSA (A^2^)	Lipinski Violation
**2a**	301.34	1.29	0	3	3	56.47	0
**2b**	335.79	1.80	0	3	3	56.47	0
**2c**	326.35	0.66	0	4	3	80.26	0
**2d**	315.37	1.54	0	3	3	56.47	0
**2e**	319.33	1.68	0	4	3	56.47	0
**2f**	380.24	1.92	0	3	3	56.47	0
**2g**	369.34	2.16	0	6	4	56.47	0
**2h**	346.34	0.41	0	5	4	102.29	0
Cisplatin	298.03	—	—	—	0	52.04	—
**5-FU**	130.08	−0.32	2	3	0	65.72	0
Rule of Lipinski	≤500	≤5	≤5	≤10	≤10	≤140	≤1

Human Intestinal Absorption (HIA) refers to the process by which orally administered drugs are absorbed from the intestine. All compounds tested showed high absorption rates between 92% and 98%, indicating they are well-absorbed and can be taken up by the human intestine. To evaluate a compound’s *in vitro* intestinal permeability relative to human enterocytes and to identify transporter and efflux proteins, Caco-2 cell permeability (CCP) is often used as a model. Caco-2 cells are derived from a human colon epithelial cancer line. The compounds tested demonstrated positive permeability in cell culture (CCP), indicating moderate absorption. Madin-Darby canine kidney (MDCK) cells were also used to evaluate the human intestinal barrier and drug uptake efficiency, with all compounds showing favorable MDCK permeability values ranging from 0.23 to 36.06 nm/s. P-glycoprotein (P-gp) serves as a significant barrier to effective drug delivery by expelling toxins and foreign substances from cells. To enhance the transport of therapeutic agents, inhibiting efflux pumps is necessary. The results indicated that only compounds **2e** and **2a** were not affected by P-gp inhibition. Skin permeability (SP) is an important factor for transdermal drug delivery, as drugs must distribute through the intraepithelial lipid substance, which is the primary feature for skin absorption. All compounds showed negative skin permeability values, suggesting that transdermal administration is not required. Plasma protein binding (PPB) plays a significant role in determining the duration of a drug’s presence in the body and may impact its therapeutic effectiveness. The extent of binding to plasma proteins significantly impacts a drug’s pharmacodynamic and pharmacokinetic properties, with values below 90% classified as low and those at or above 90% as high. The PPB percentages for all compounds were below 90%. The capacity of therapeutic agents to penetrate the blood-brain barrier (BBB) is a critical factor in evaluating their potential side effects and toxicity. All tested compounds showed positive BBB penetration values, indicating they can cross the barrier, but their values were below 1 (CBrain/Cblood <1), suggesting they are inactive in the central nervous system (CNS) (Table 5).

**TABLE 5:** In silico ADME profile of the pyrimidine-morpholin derivatives (***2a*-*2h***).

Entry	HIA%	Caco2 (nm/sec)	MDCK (nm/sec)	P-gp inhibition	*In vitro* skin permeability (logKp, cm h^−1^)	PPB%	BBB
**2a**	98.21	30.87	16.47	non	−4.06	45.18	1.10
**2b**	97.72	37.98	10.64	Inhibitor	−4.11	72.75	1.07
**2c**	98.20	20.12	16.14	Inhibitor	−4.07	52.03	0.03
**2d**	98.13	31.31	29.82	Inhibitor	−4.04	59.12	1.36
**2e**	98.20	43.56	19.71	non	−4.28	64.81	0.14
**2f**	97.44	43.36	0.30	Inhibitor	−4.01	74.77	0.31
**2g**	98.11	27.59	0.23	Inhibitor	−2.91	75.20	0.03
**2h**	92.45	14.03	36.06	Inhibitor	−4.01	59.60	0.02
Cisplatin	93.58	19.88	0.52	non	−4.50	73.45	0.38
**5-FU**	75.93	17.25	0.68	non	−5.02	8.31	0.20

Toxicity prediction is a crucial aspect of the drug discovery process, as some drug candidates may fail clinical trials due to their toxicity. The toxicity profile of the designed compounds is shown in Table 6. Mutagenicity and carcinogenicity of a compound can be predicted by the Ames test and a 2-year carcinogenicity evaluation in mice. Based on the results of the Ames test, all compounds were mutagenic. The carcinogenicity test on mice showed that compounds **2c**, **2e**, **2f**, and **2h** were carcinogenic, while the rest tested negative and were not carcinogens. The cardiac toxicity was determined using the human ether-a-go-go-related gene, HERG-inhibition assay. The blockage of the HERG leads to fatal ventricular tachyarrhythmia due to the lengthening of the QT interval. All compounds exhibited a moderate risk of HERG inhibition.

**TABLE 6:** Toxicity prediction of the pyrimidine-morpholine derivatives (**2a-2h**).

Entry	Ames_test	Carcino_Mouse	HERG_Inhibition	Algae_at
**2a**	Mutagen	Negative	Medium-risk	0.17
**2b**	Mutagen	Negative	Medium-risk	0.09
**2c**	Mutagen	Positive	Medium-risk	0.13
**2d**	Mutagen	Negative	Medium-risk	0.10
**2e**	Mutagen	Positive	Medium-risk	0.13
**2f**	Mutagen	Positive	Medium-risk	0.07
**2g**	Mutagen	Negative	Medium-risk	0.07
**2h**	Mutagen	Positive	Medium-risk	0.15
Cisplatin	Non-mutagen	Negative	Medium-risk	0.19
**5-FU**	Mutagen	Positive	Medium-risk	0.43

## Conclusion

A novel series of morpholine-pyrimidine hybrid compounds was created as potential agents to inhibit cell growth and synthesized. The chemical structures were characterized using FT-IR, ^1^HNMR, and ^13^CNMR spectroscopy. The antiproliferative effects were evaluated on two human cancerous cell lines, SW480 and MCF-7. When compared to each other and to cisplatin and 5-FU as positive controls, compound 2g demonstrated the highest antiproliferative activity against the SW480 cell line, with an IC_50_ value of (IC_50_ = 5.1 ± 2.12) μM, in contrast to (IC_50_ = 16.1 ± 1.1) μM for Cisplatin and 5-FU, which had an IC_50_ of (IC50 = 4.9 ± 0.83) μM. Structure-activity relationship studies suggest that the presence of a 4-CF_3_ group on the phenyl ring and an electron-withdrawing group like cyano on the benzyl part significantly enhances antiproliferative activity. The molecular docking results supported the biological activity findings. Overall, compound **2g** appears to be a propitious candidate for subsequent *in vivo* and *in vitro* anticancer research. Additionally, DFT analysis indicated that compound **2g** is more stable both thermodynamically and kinetically than compound **2h**.

## Data Availability

The original contributions presented in the study are included in the article/Supplementary Material, further inquiries can be directed to the corresponding authors.
