# Supplementary material for: Design, synthesis, in silico studies and antiproliferative evaluation of some novel hybrids of pyrimidine-morpholine
Source: Front Chem. 2025 Feb 28;13:1537261. doi: 10.3389/fchem.2025.1537261 (PMC11906455; doi:10.3389/fchem.2025.1537261)
Supplement: Supplementary file 1 [file DataSheet1.docx]

**Design, Synthesis, *in silico* studies and Antiproliferative Evaluation of Some Novel** **Pyrimidine-Morpholine Derivatives**

Elaheh Ataollahi^1a^, Leila Emami^1a^, Al-Anood Mohammad Al-Dies^2^, Fateme Zare^1^, Alireza Poustforoosh^3^, Mina Emami^1^, Fateme Saadat^4^, Fateme Motamen^4^, Zahra Rezaei^5*^, Soghra Khabnadideh^1,5^[[1]](#footnote-1)^^

*^1^Pharmaceutical Sciences Research Center, Shiraz University of Medical Sciences, Shiraz, Iran.*

*^2^Chemistry Deparment, UMM Al-Qura University, Al Qunfudah University College, Saudi Arabia*

*^3^Medicinal and Natural Products Chemistry Research Center, Shiraz University of Medical Sciences, Shiraz, Iran*

*^4^Student Research Committee, School of Pharmacy, Shiraz University of Medical Sciences, Shiraz, Iran*

*^5^Department of Medicinal Chemistry, Faculty of Pharmacy, Shiraz University of Medical Sciences, Shiraz, I.R. Iran.*


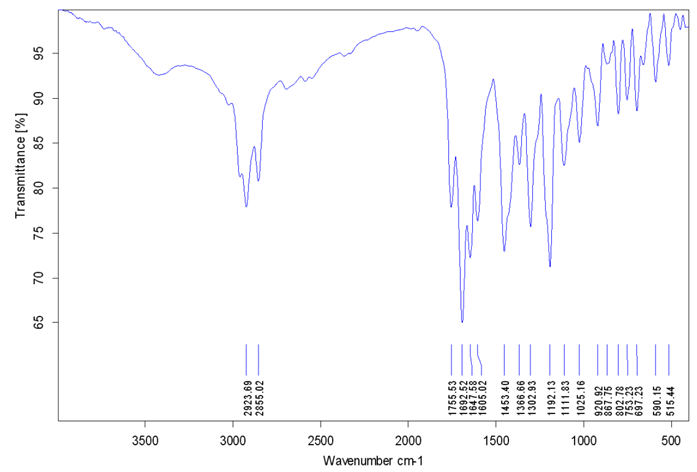


**Figure S1**: FT-IR spectrum of **2a**


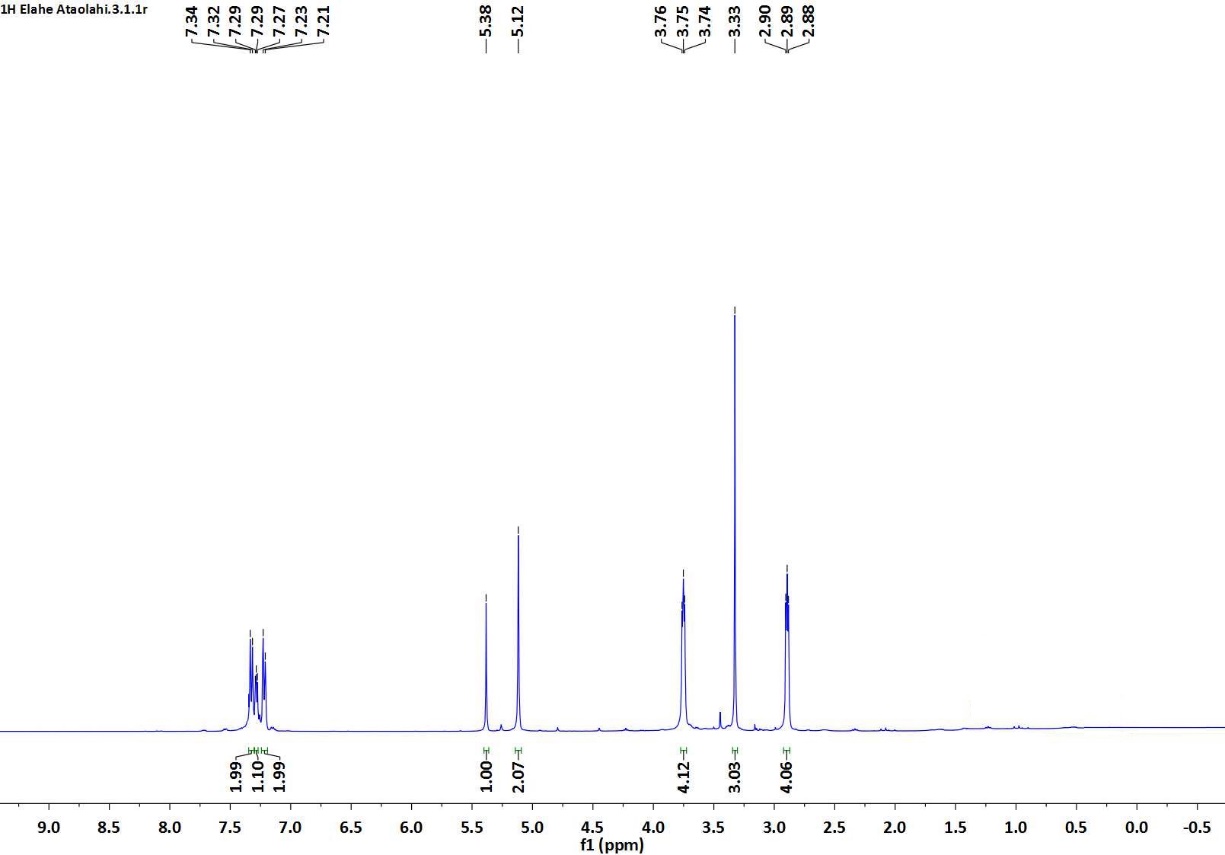


**Figure S2**: ^1^HNMR spectrum of **2a**


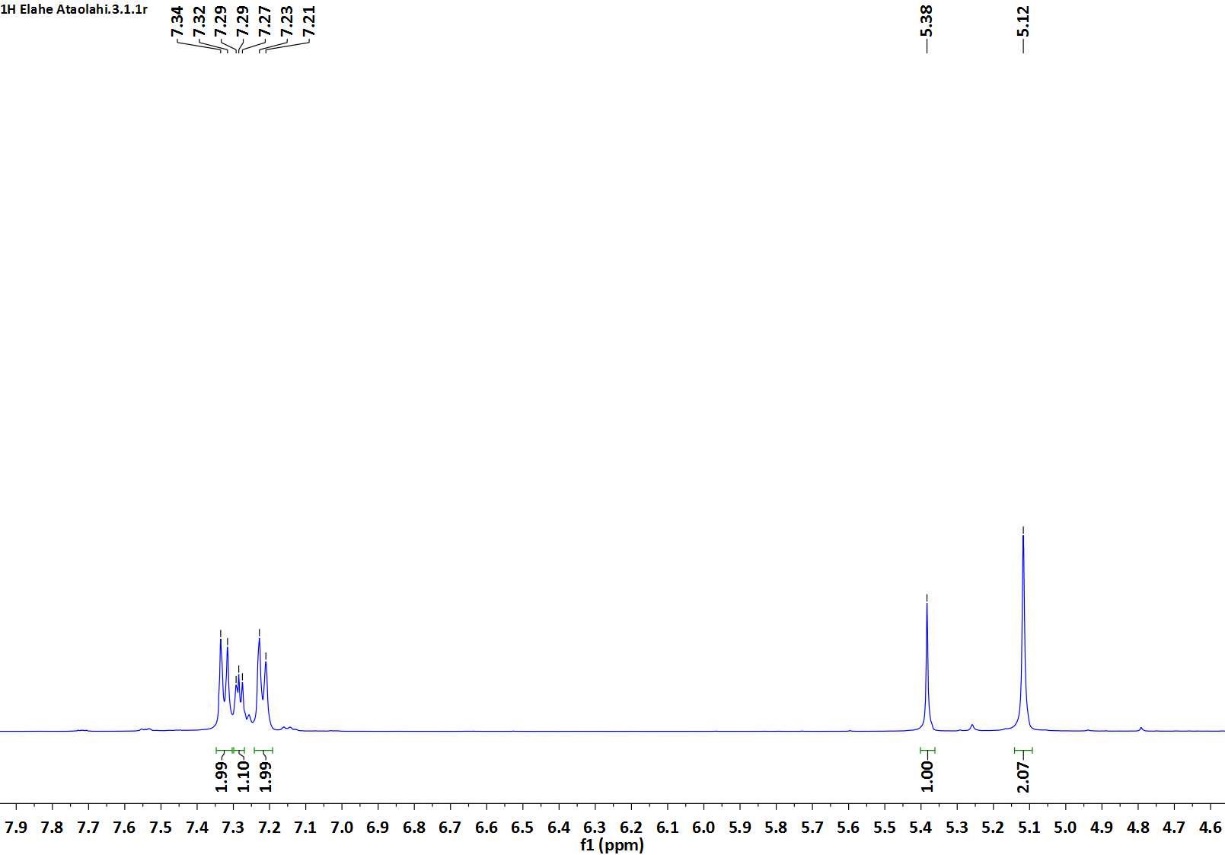


**Figure S3**: ^1^HNMR aromatic expand spectrum of **2a**

**
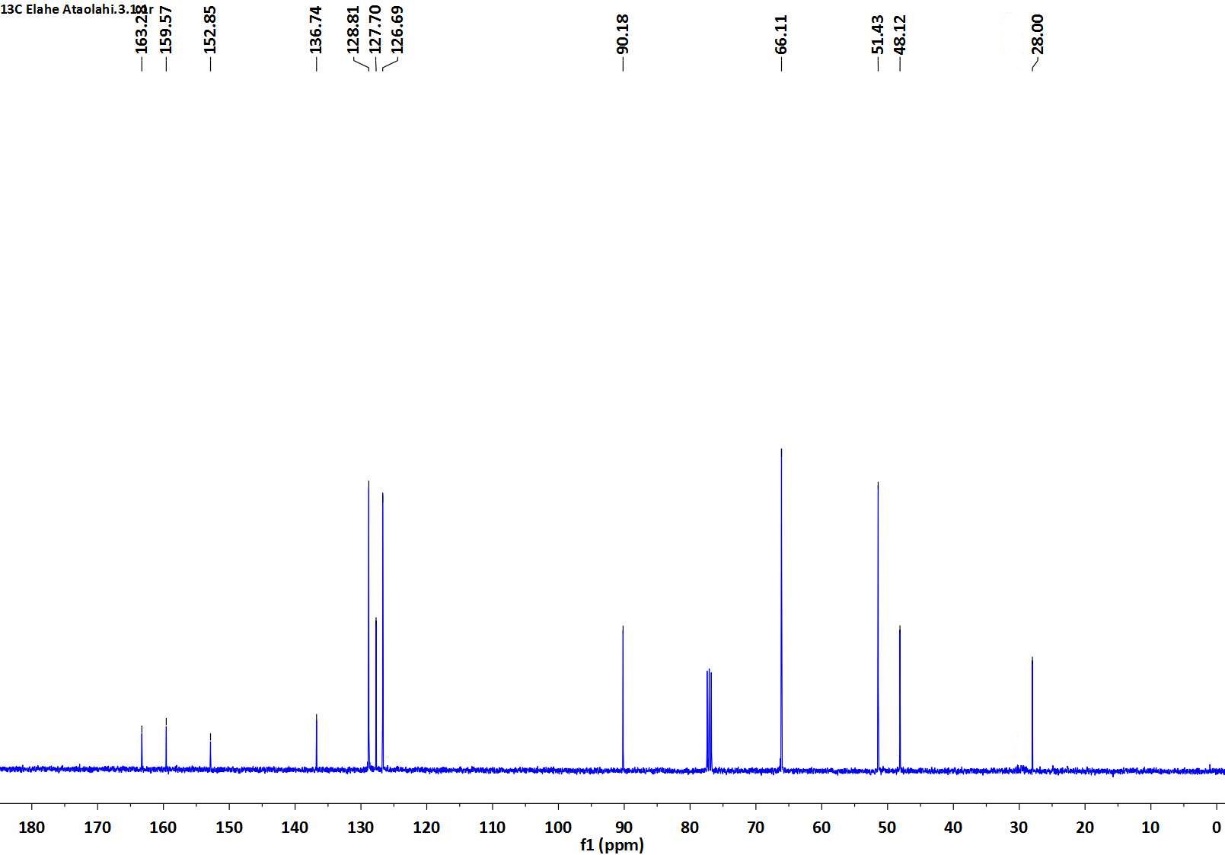
**

**Figure S4**: ^13^CNMR spectrum of **2a**


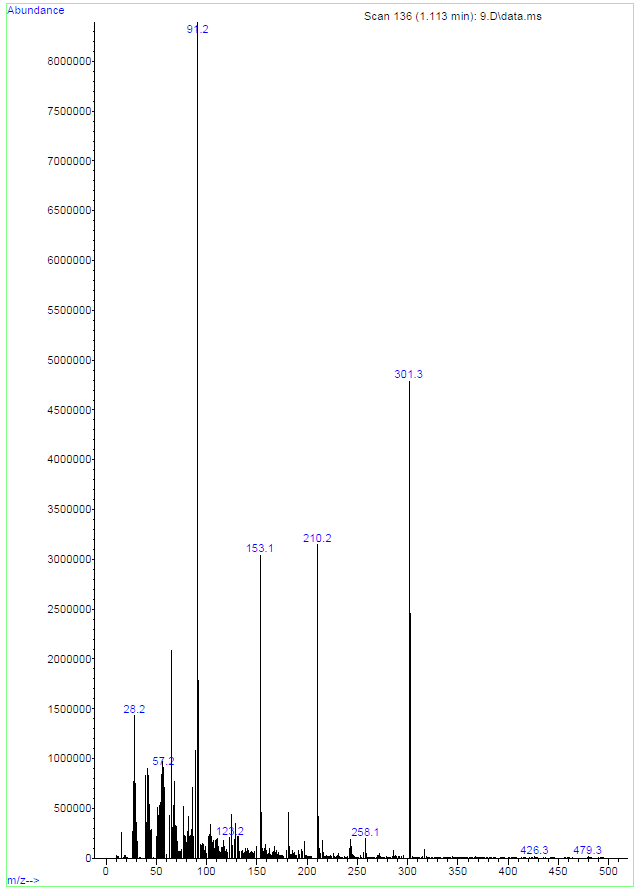


**Figure S5**: Mass spectroscopy of **2a**


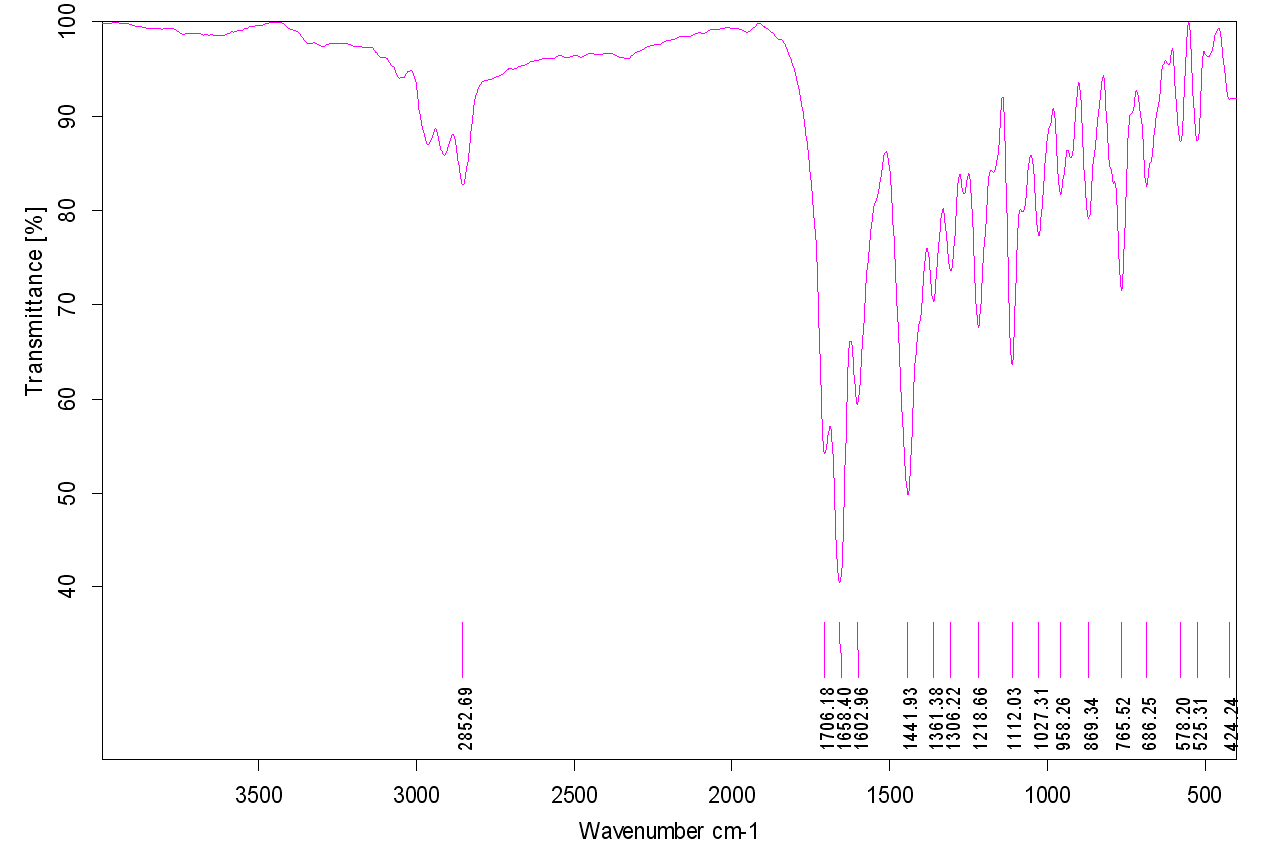


**Figure S6**: FT-IR spectrum of **2b**


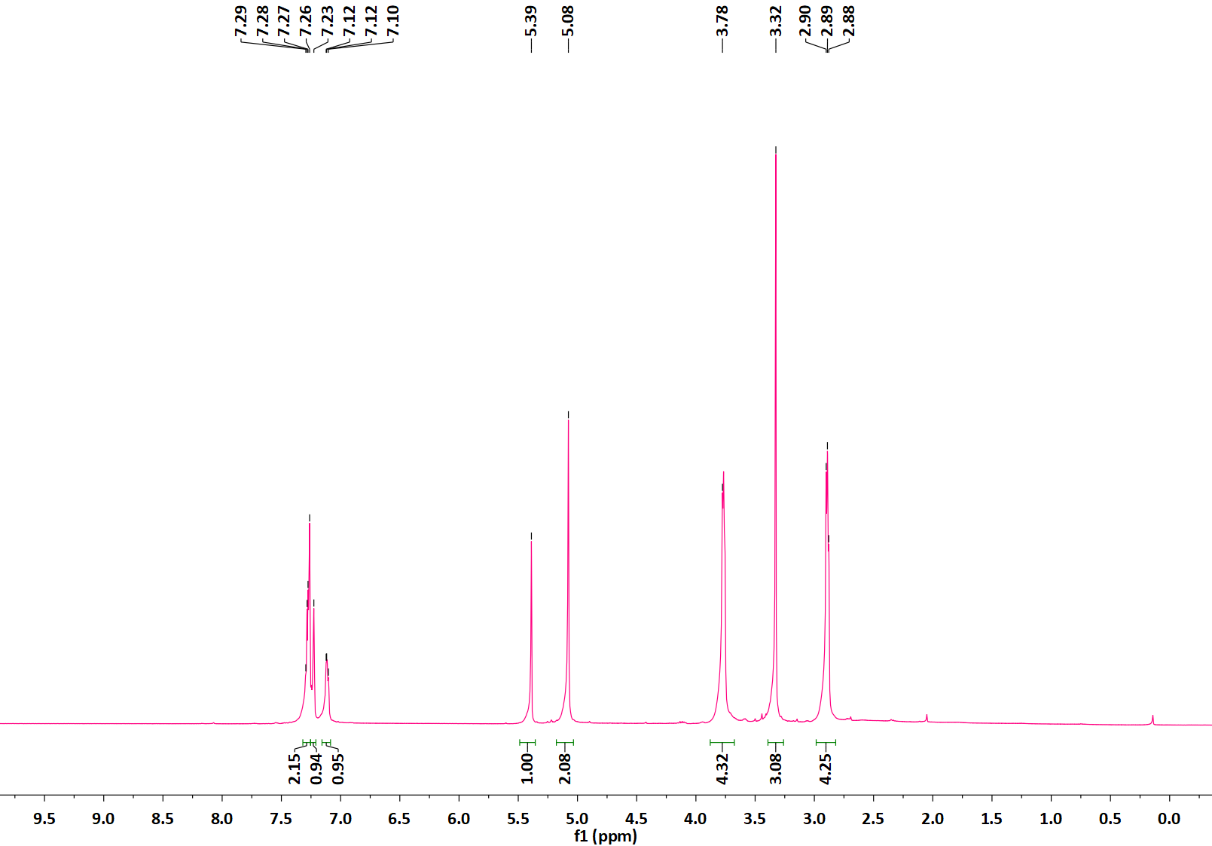


**Figure S7**: ^1^HNMR spectrum of **2b**


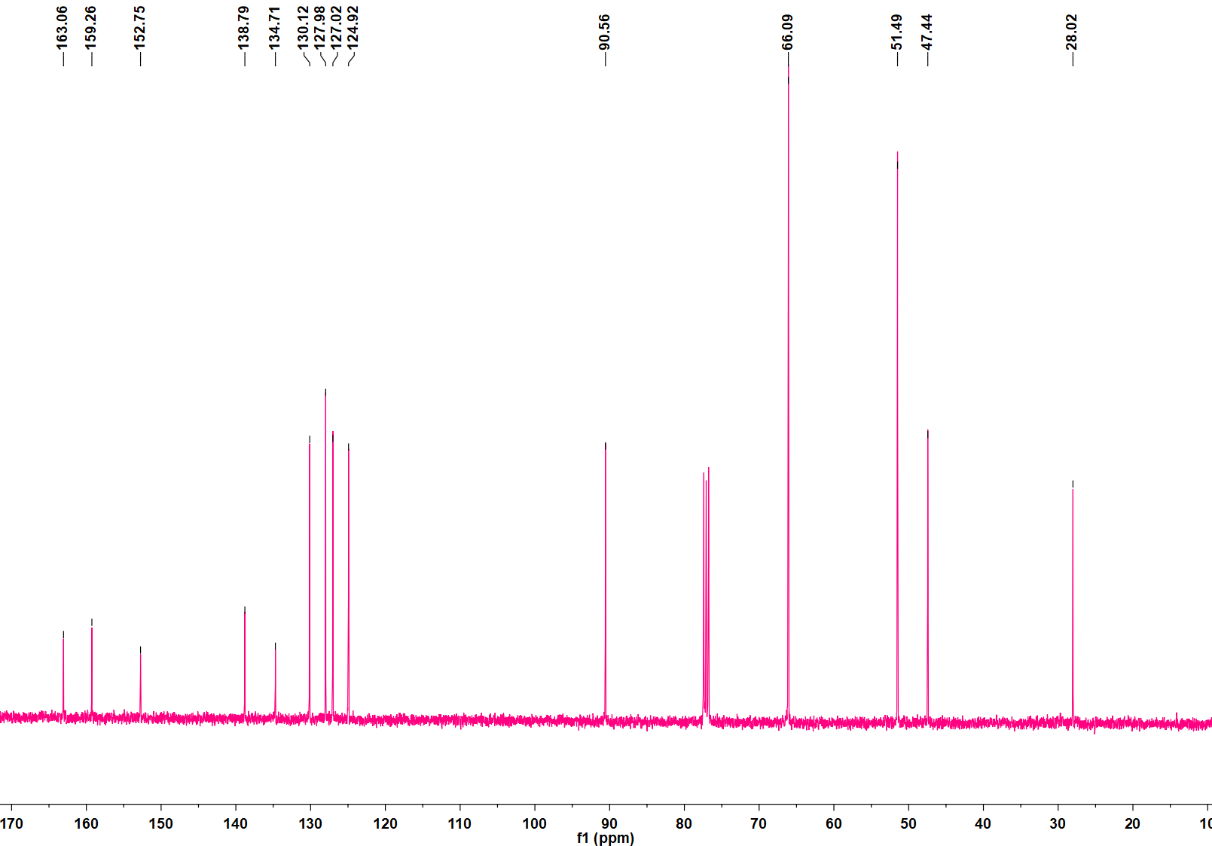


**Figure S8**: ^13^CNMR spectrum of **2b**


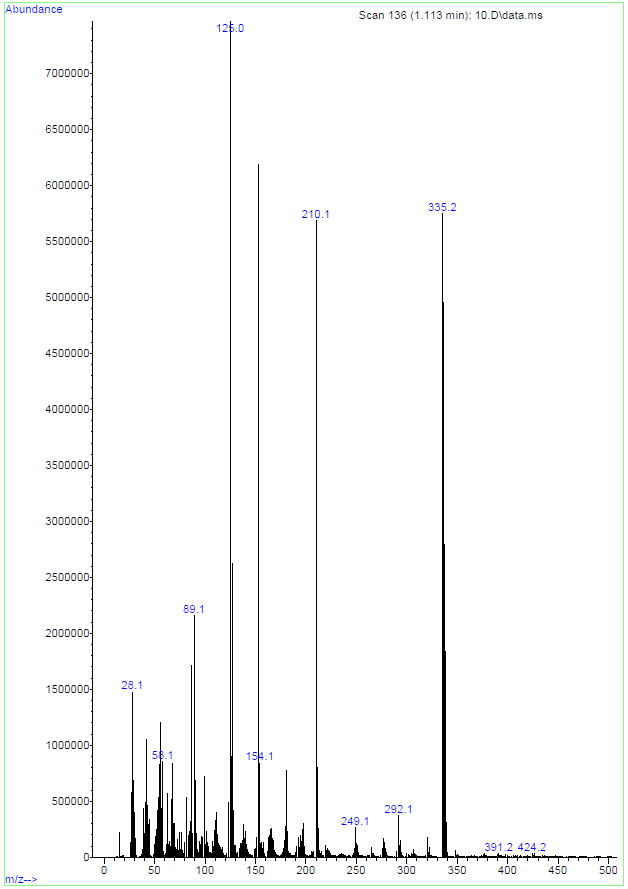


**Figure S9**: Mass spectroscopy of **2b**


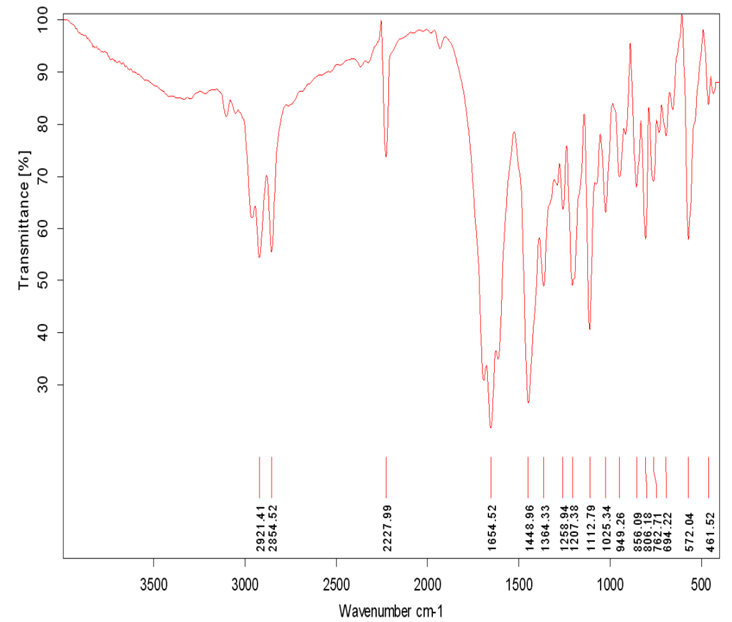


**Figure S10**: FT-IR spectrum of **2c**


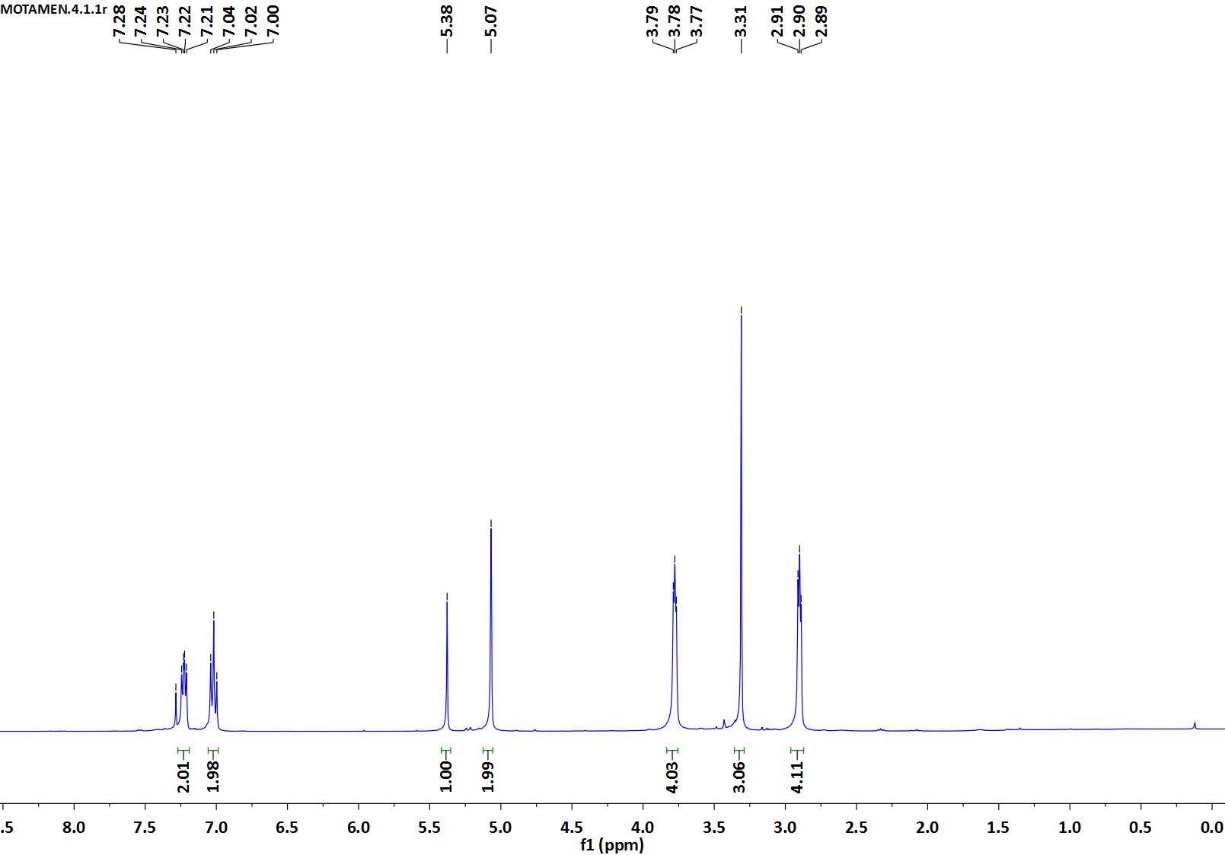


**Figure S11**: ^1^HNMR spectrum of **2c**


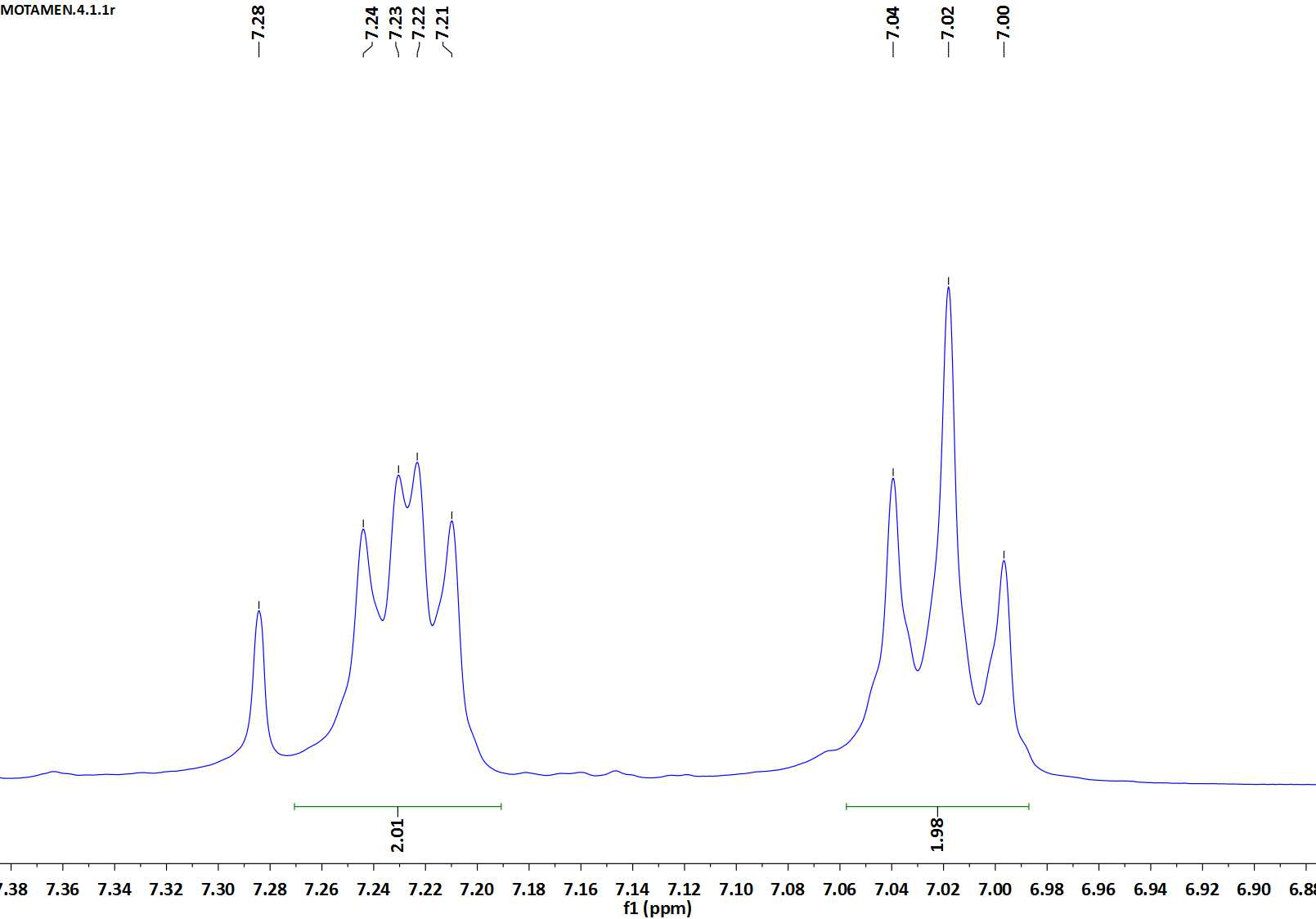


**Figure S12**: ^1^HNMR aromatic expand spectrum of **2c**


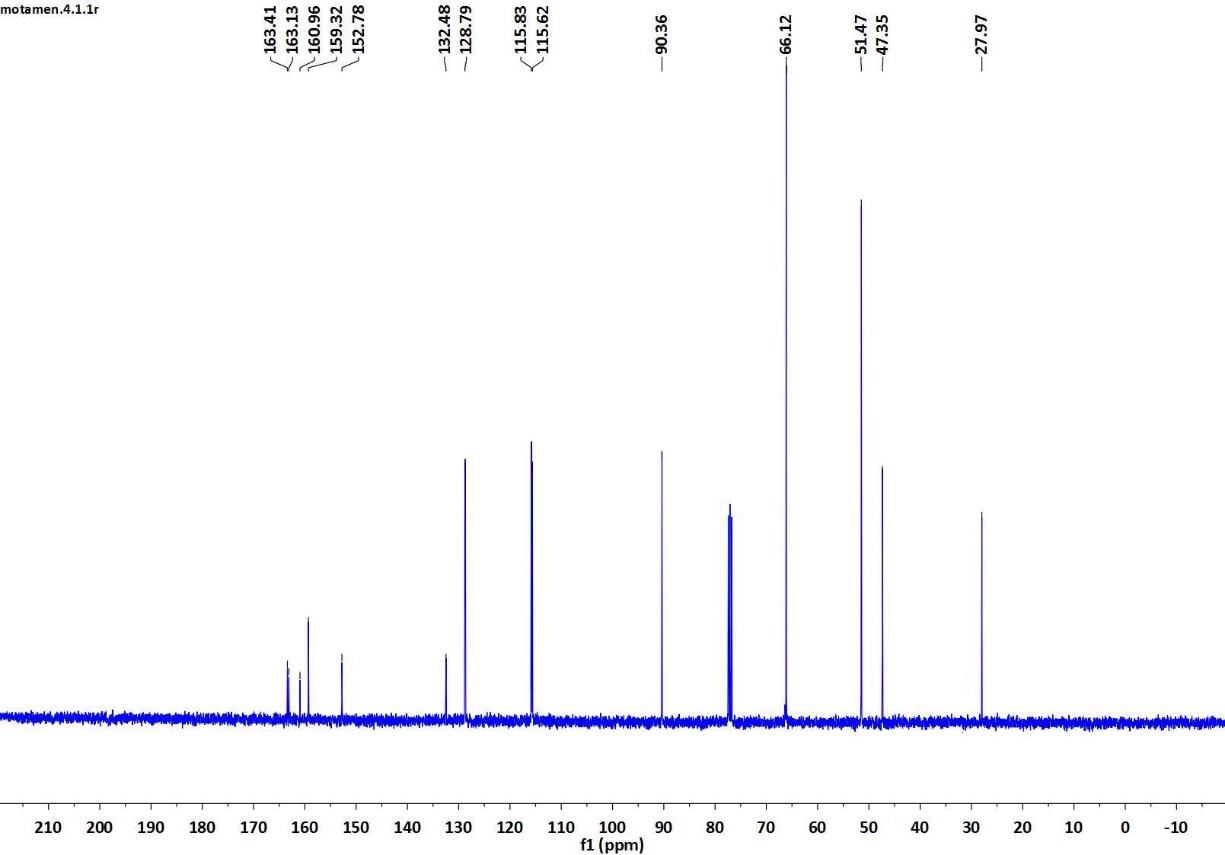


**Figure S13**: ^13^CNMR spectrum of **2c**


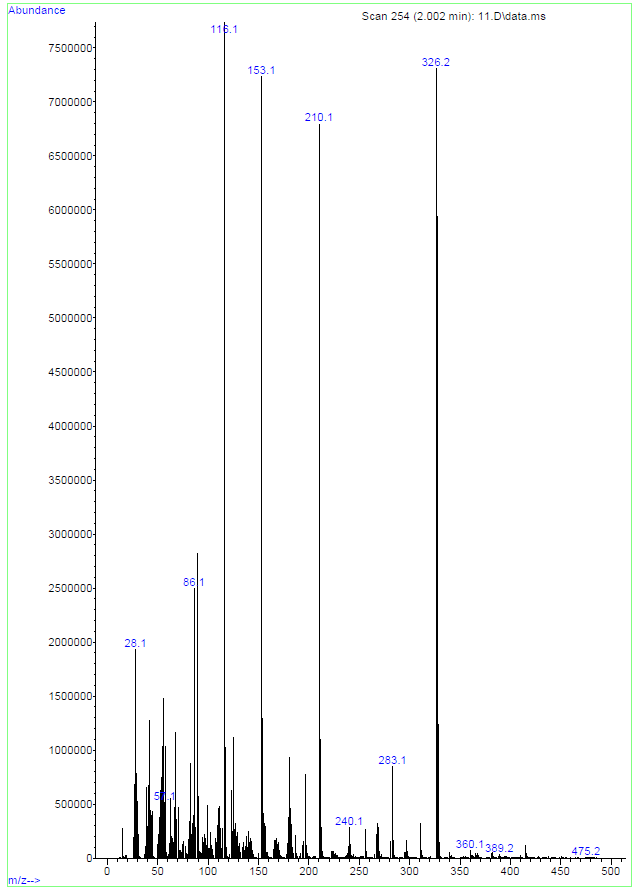


**Figure S14**: Mass spectroscopy of **2c**


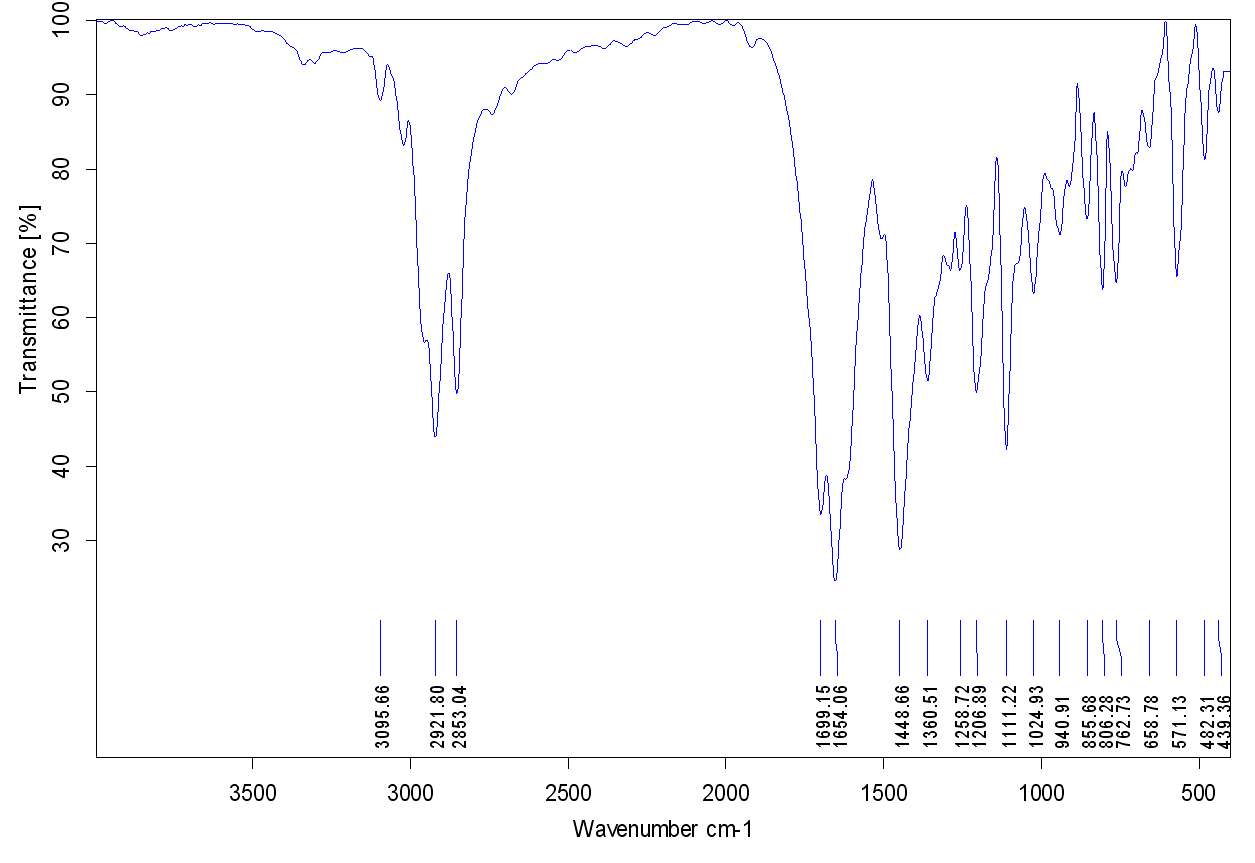


**Figure S15**: FT-IR spectrum of **2d**


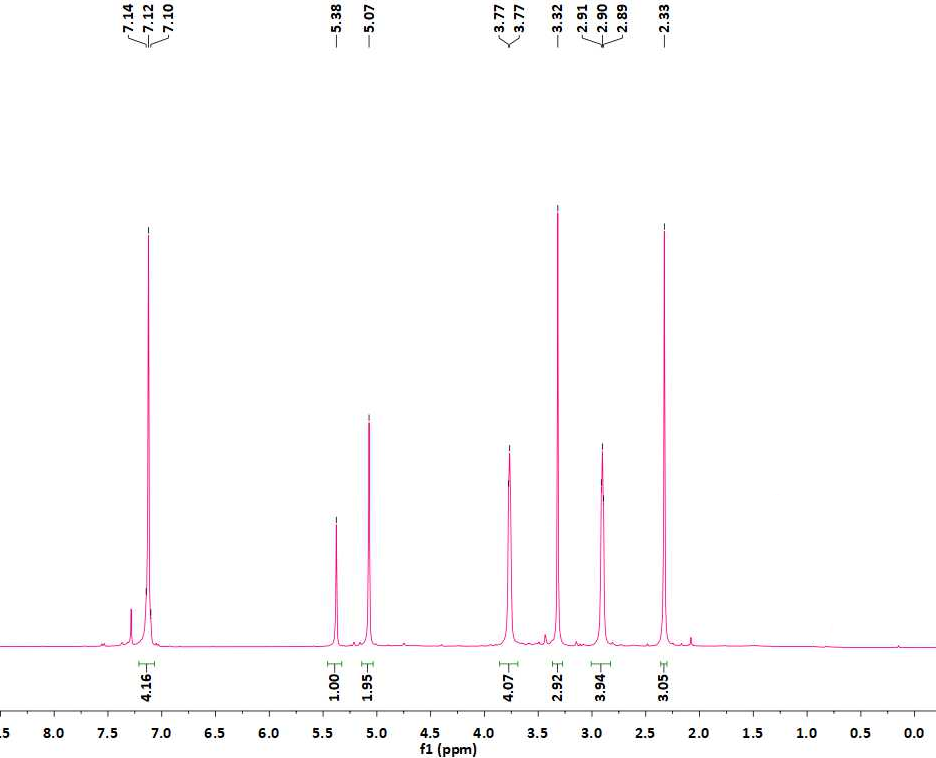


**Figure S16**: ^1^HNMR spectrum of **2d**


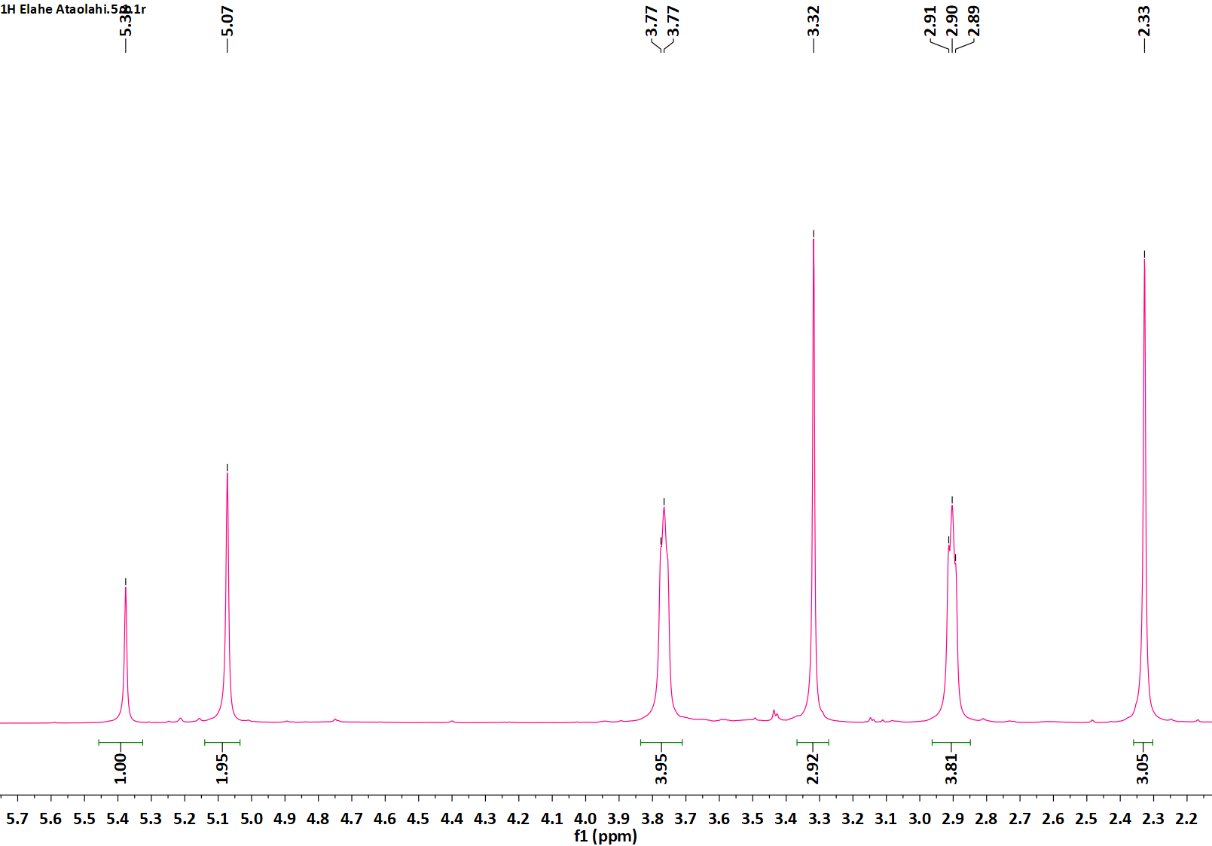


**Figure S17**: ^1^HNMR aliphatic expand spectrum of **2d**


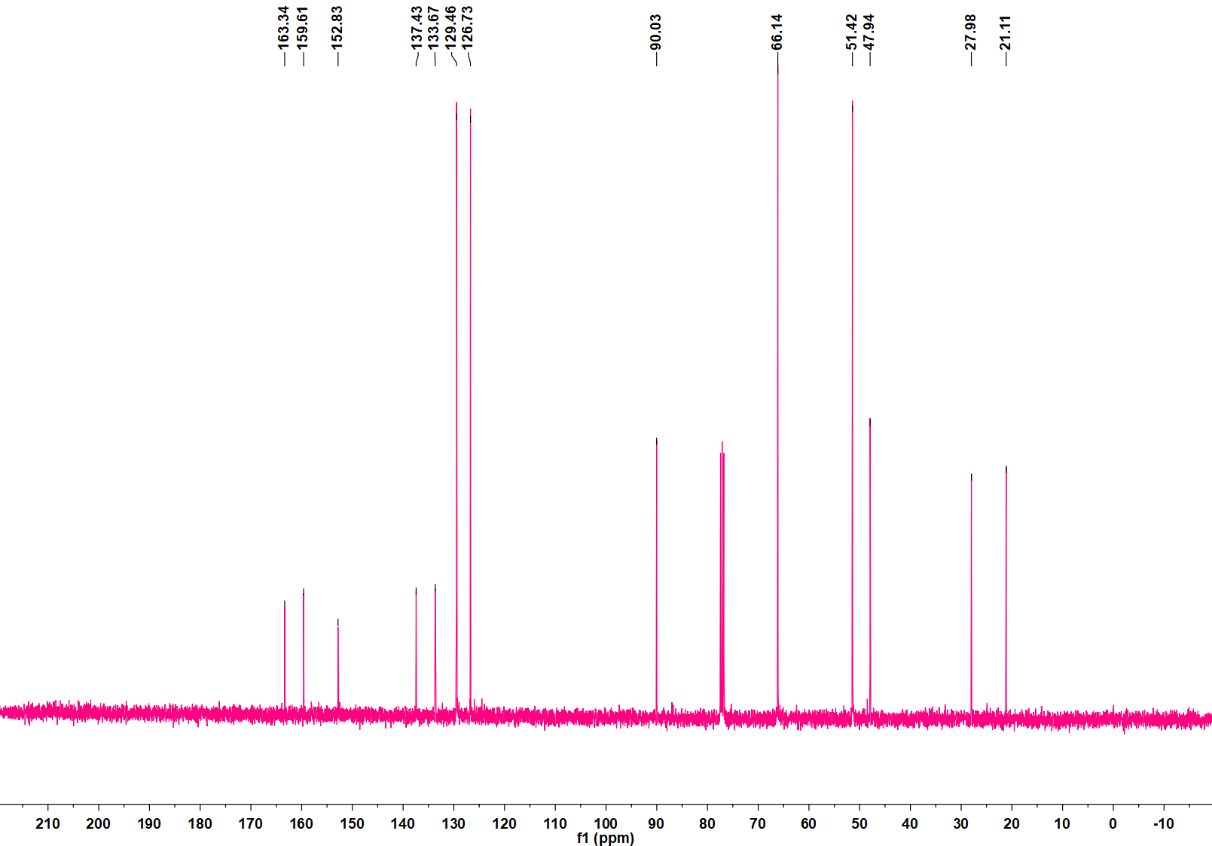


**Figure S18**: ^13^CNMR spectrum of **2d**


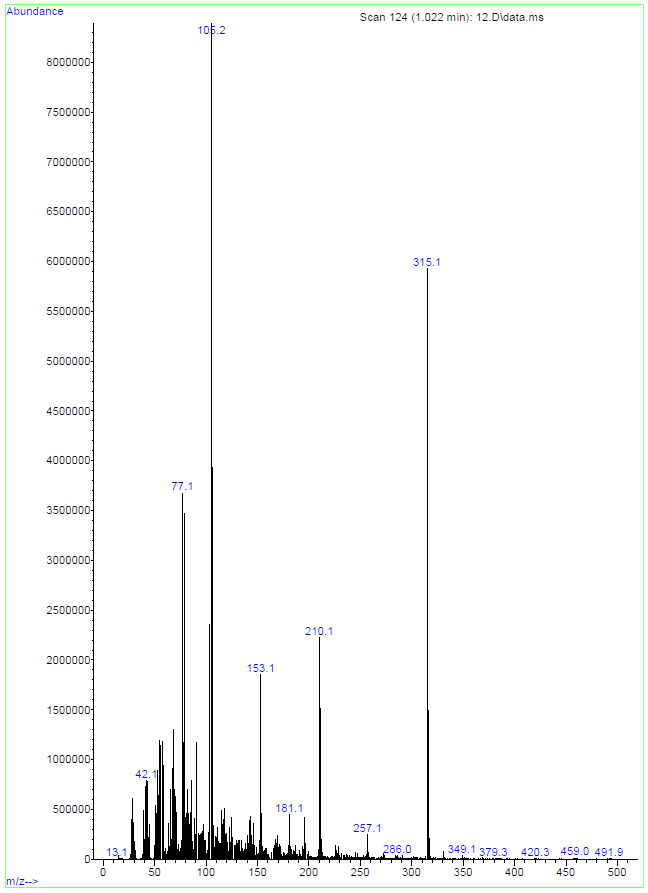


**Figure S19**: Mass spectroscopy of **2d**

**
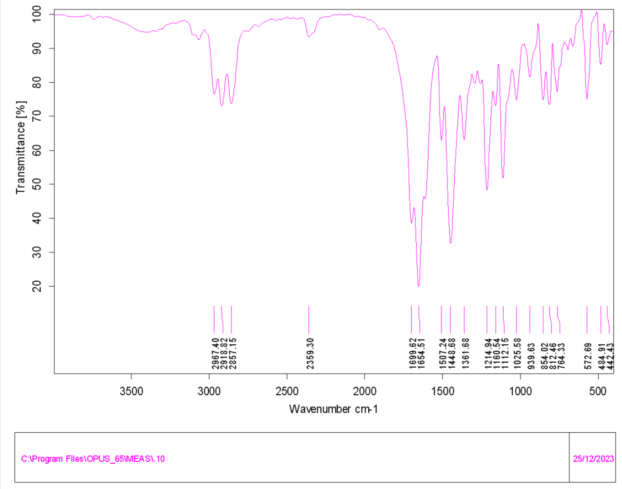
**

**Figure S20**: FT-IR spectrum of **2e**


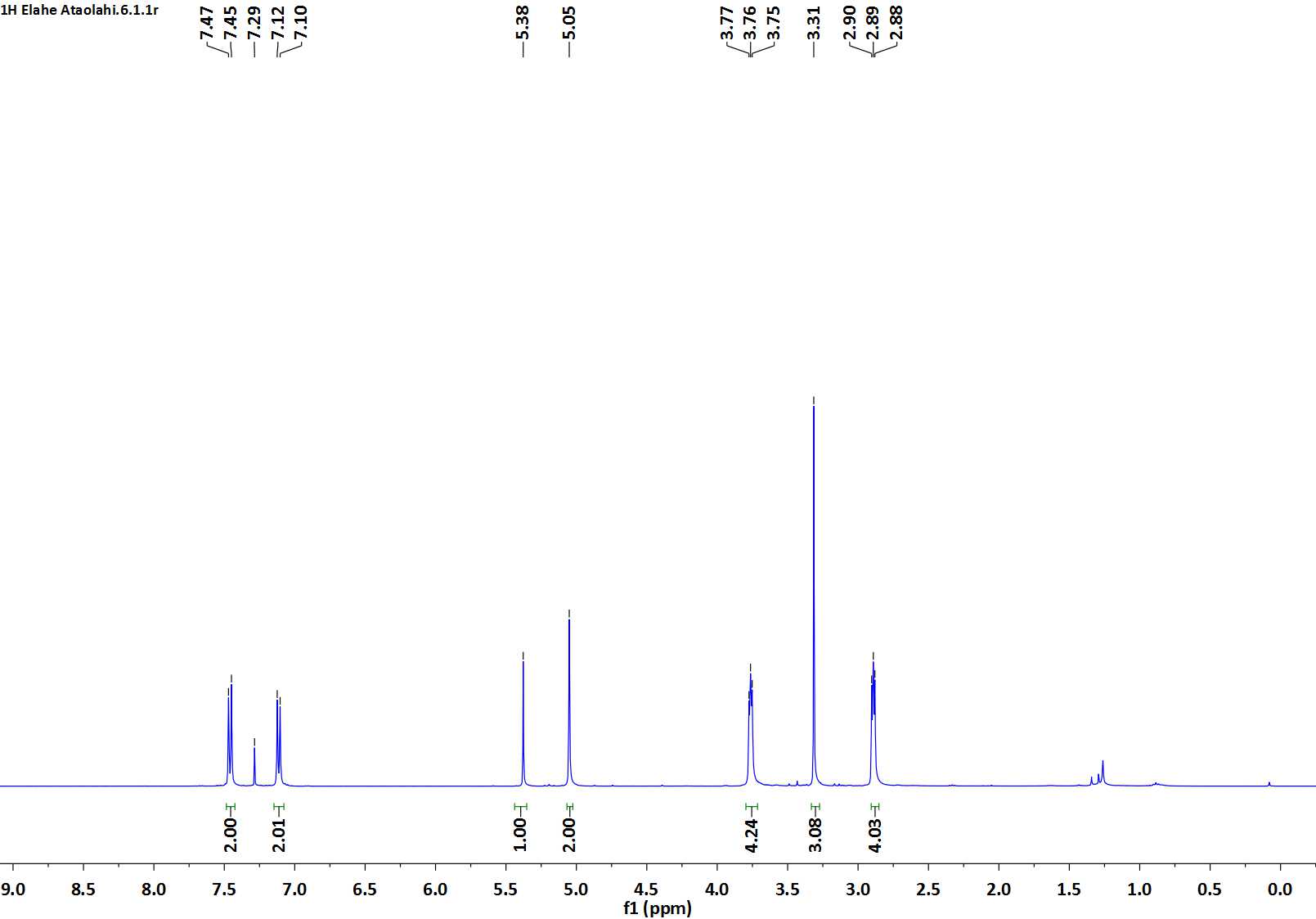


**Figure S21**: ^1^HNMR spectrum of **2e**


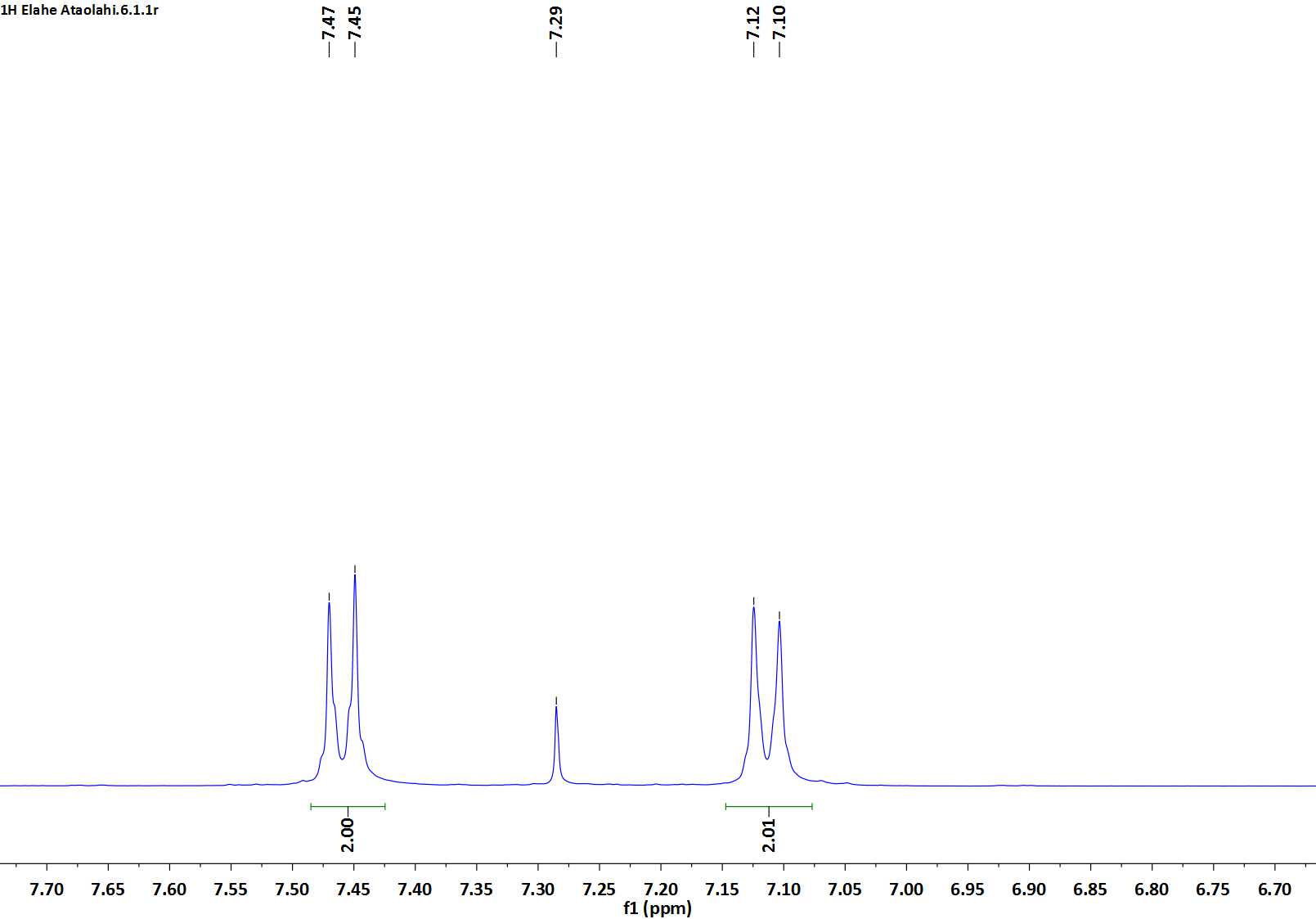


**Figure S22**: ^1^HNMR aromatic expand spectrum of **2e**


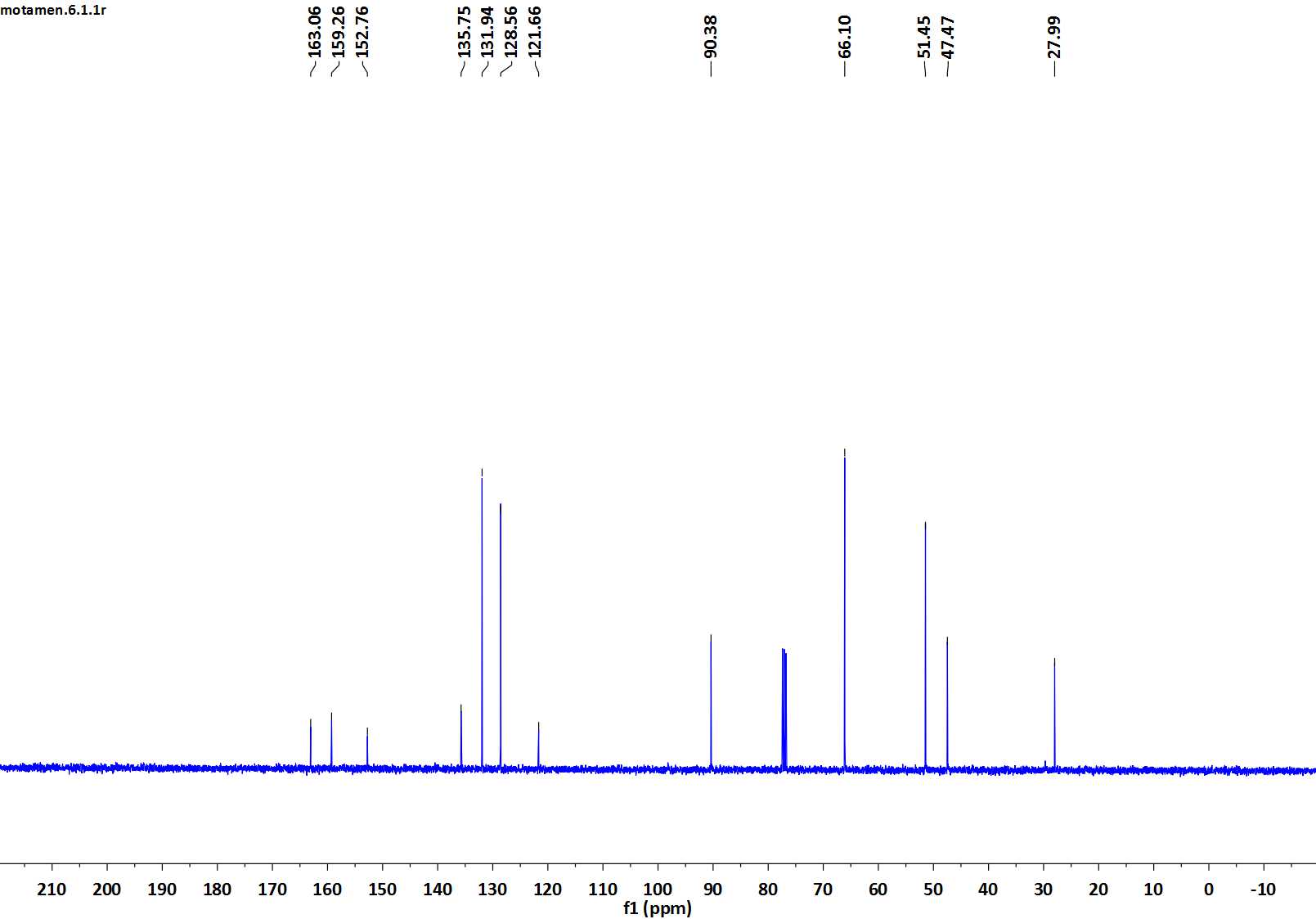


**Figure S23**: ^13^CNMR spectrum of **2e**


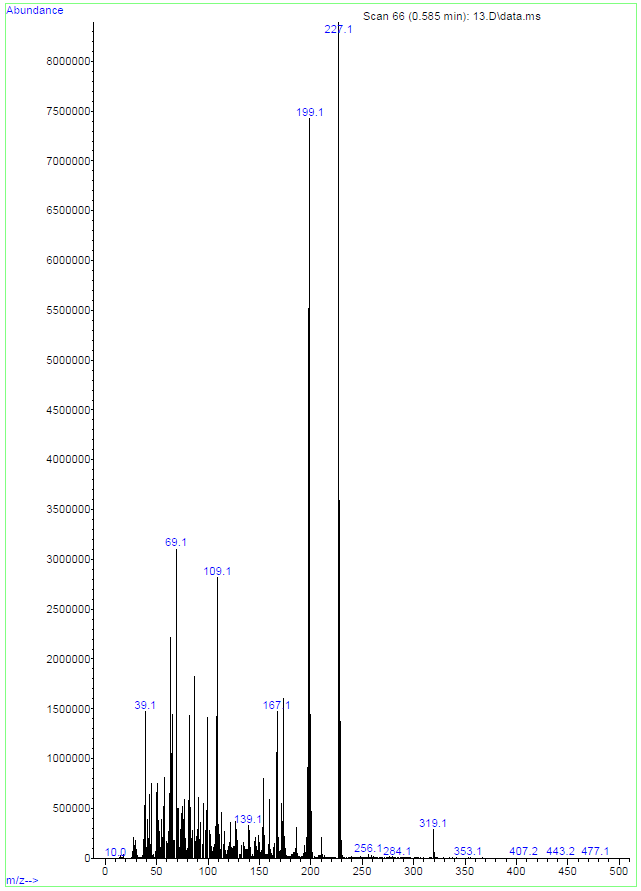


**Figure S24**: Mass spectroscopy of **2e**


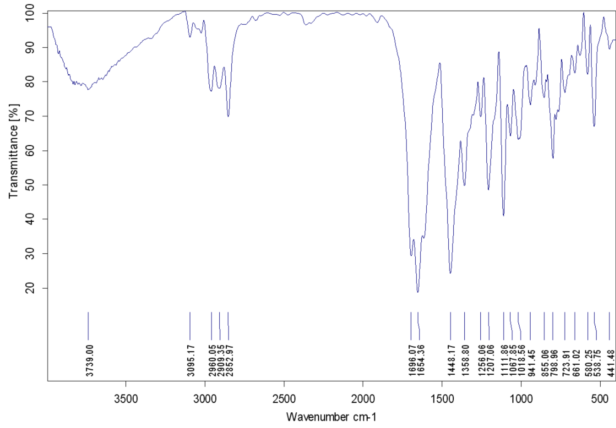


**Figure S25**: FT-IR spectrum of **2f**


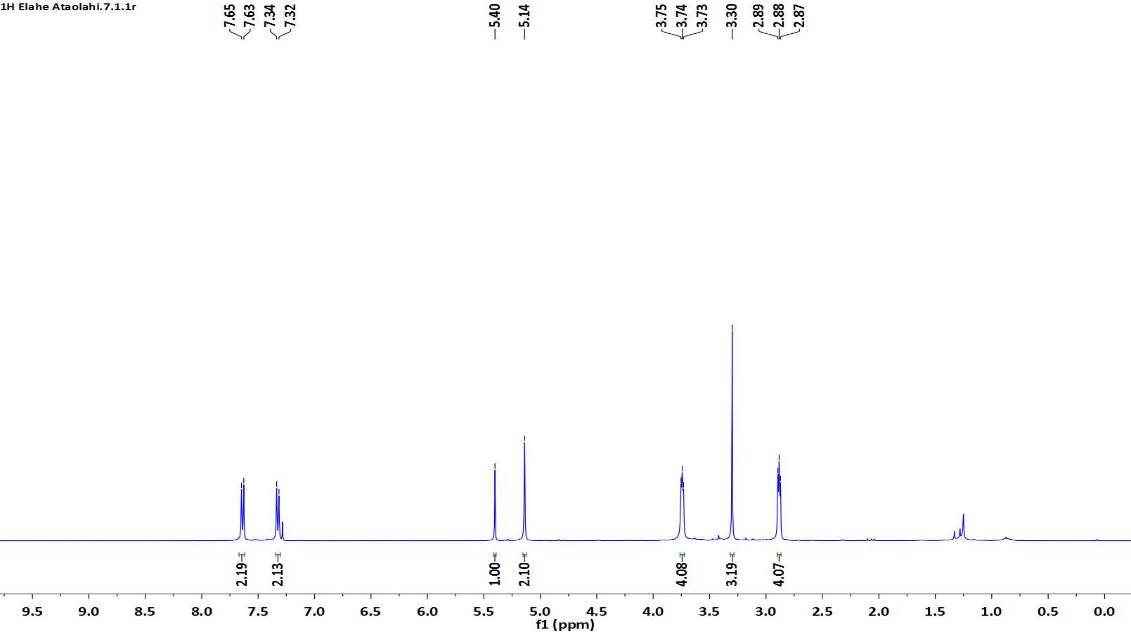


**Figure S26**: ^1^HNMR spectrum of **2f**


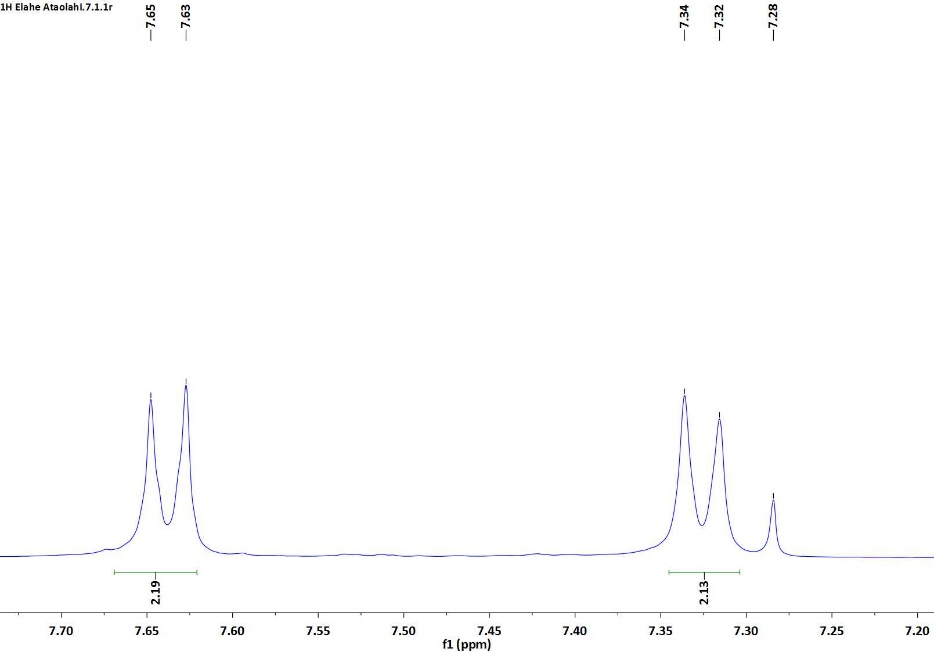


**Figure S27**: ^1^HNMR aromatic expand spectrum of **2f**


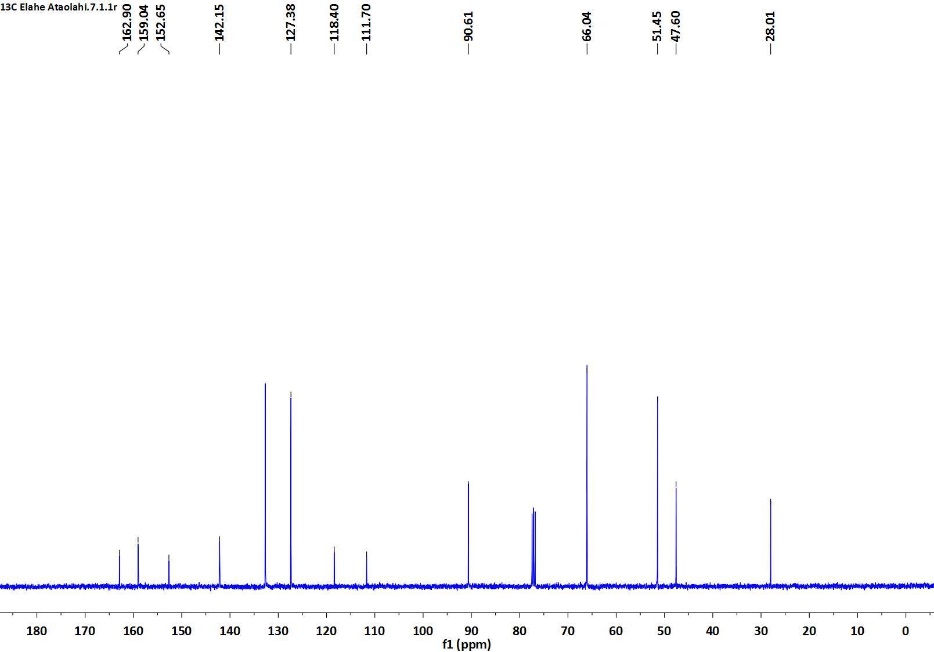


**Figure S28**: ^13^CNMR spectrum of **2f**


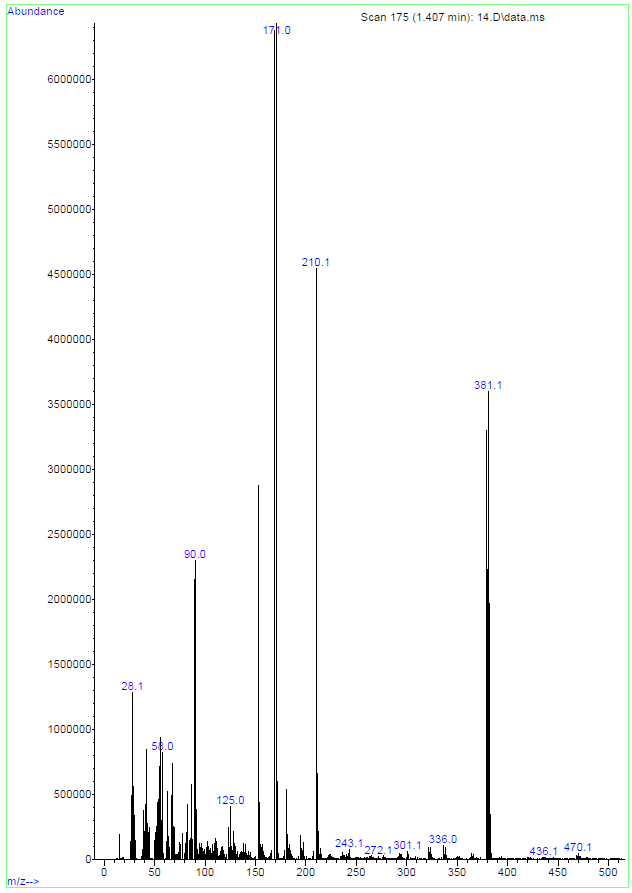


**Figure S29**: Mass spectroscopy of **2f**


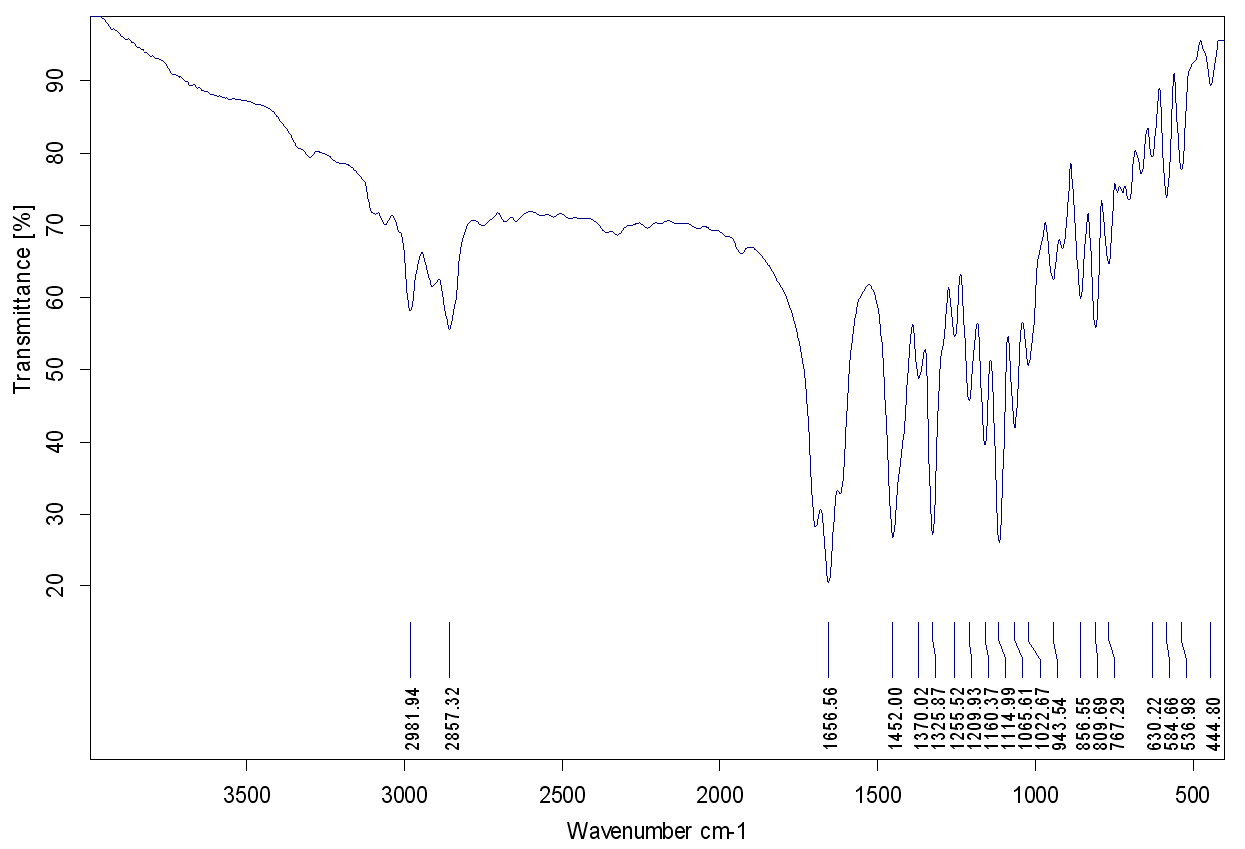


**Figure S30**: FT-IR spectrum of **2g**


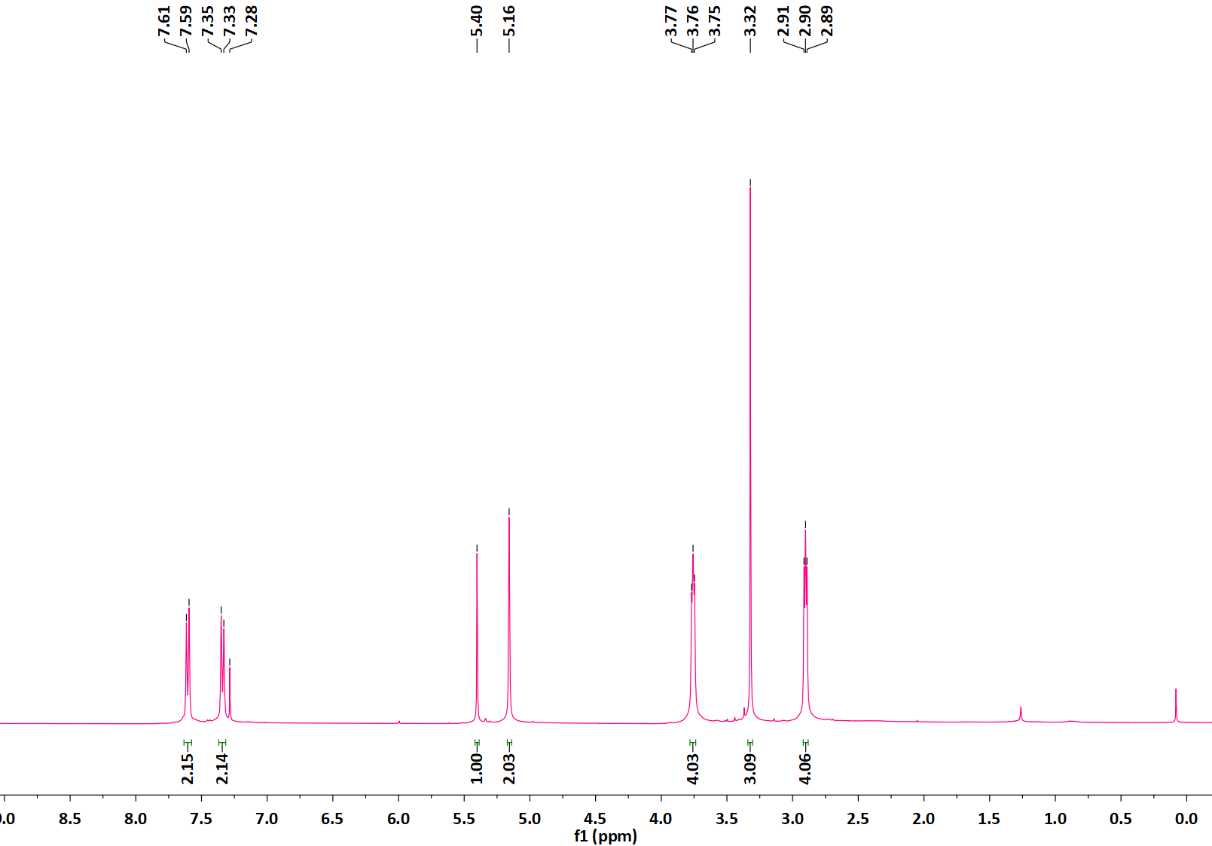


**Figure S31**: ^1^HNMR spectrum of **2g**


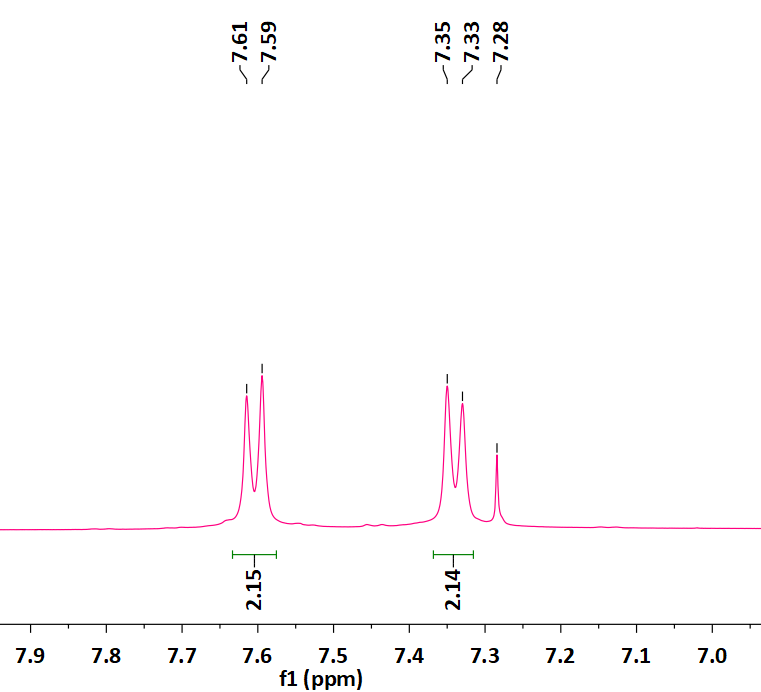


**Figure S32**: ^1^HNMR aromatic expand spectrum of **2g**


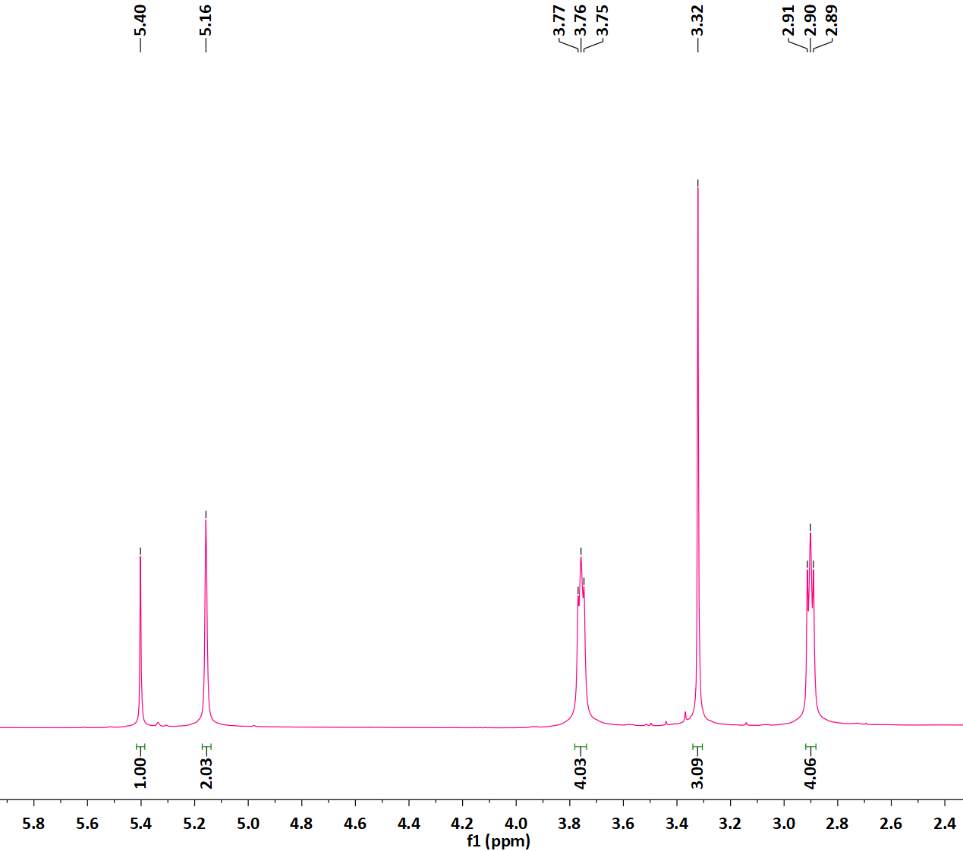


**Figure S33**: ^1^HNMR aliphatic expand spectrum of **2g**


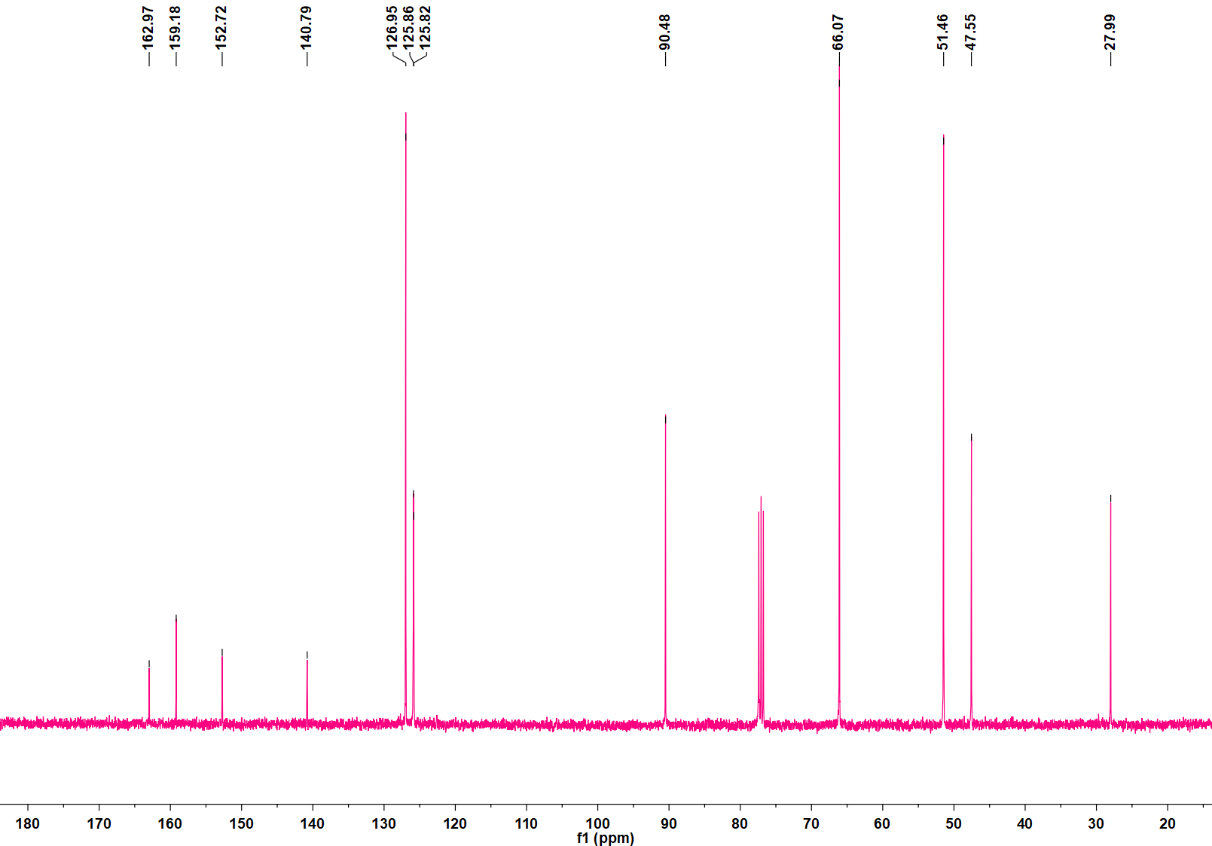


**Figure S34**: ^13^CNMR spectrum of **2g**


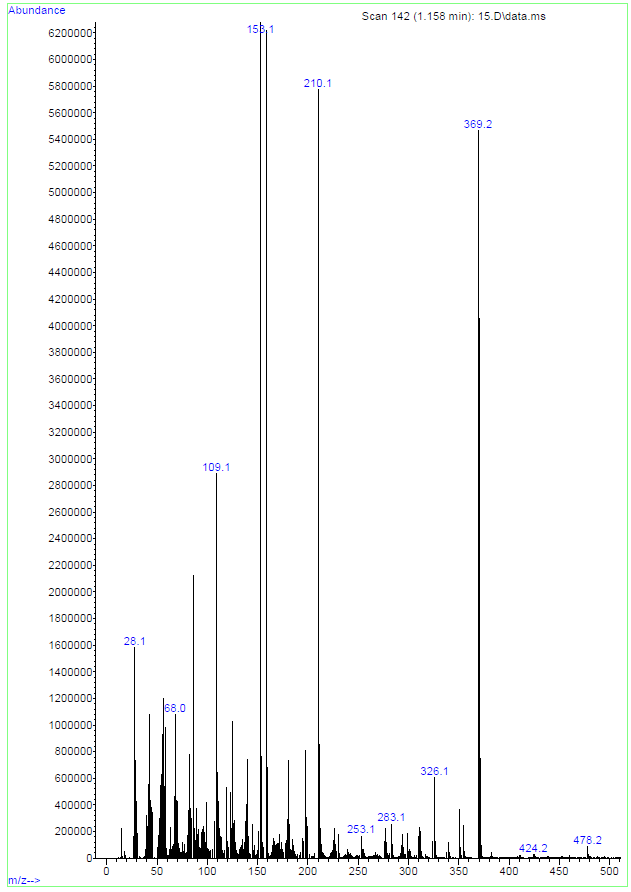


**Figure S35**: Mass spectroscopy of **2g**


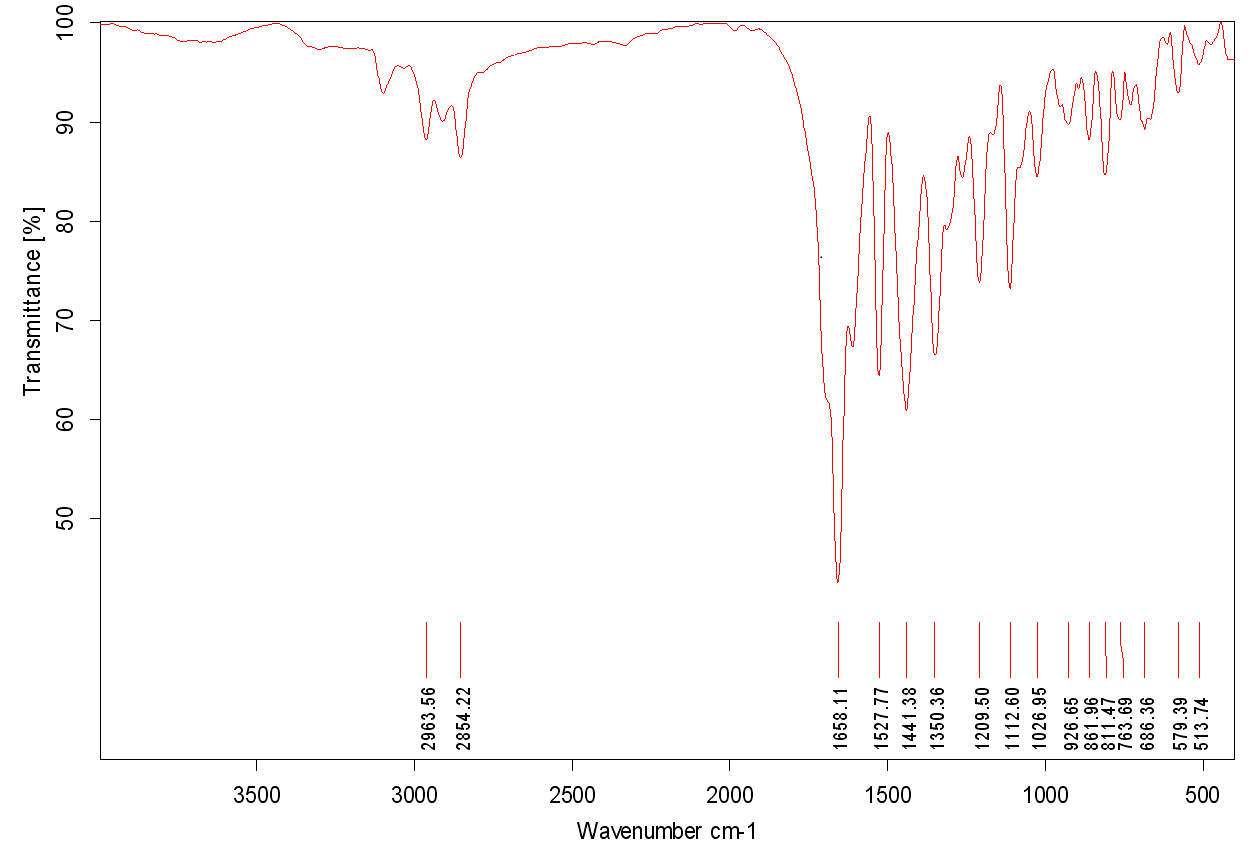


**Figure S36**: FT-IR spectrum of **2h**


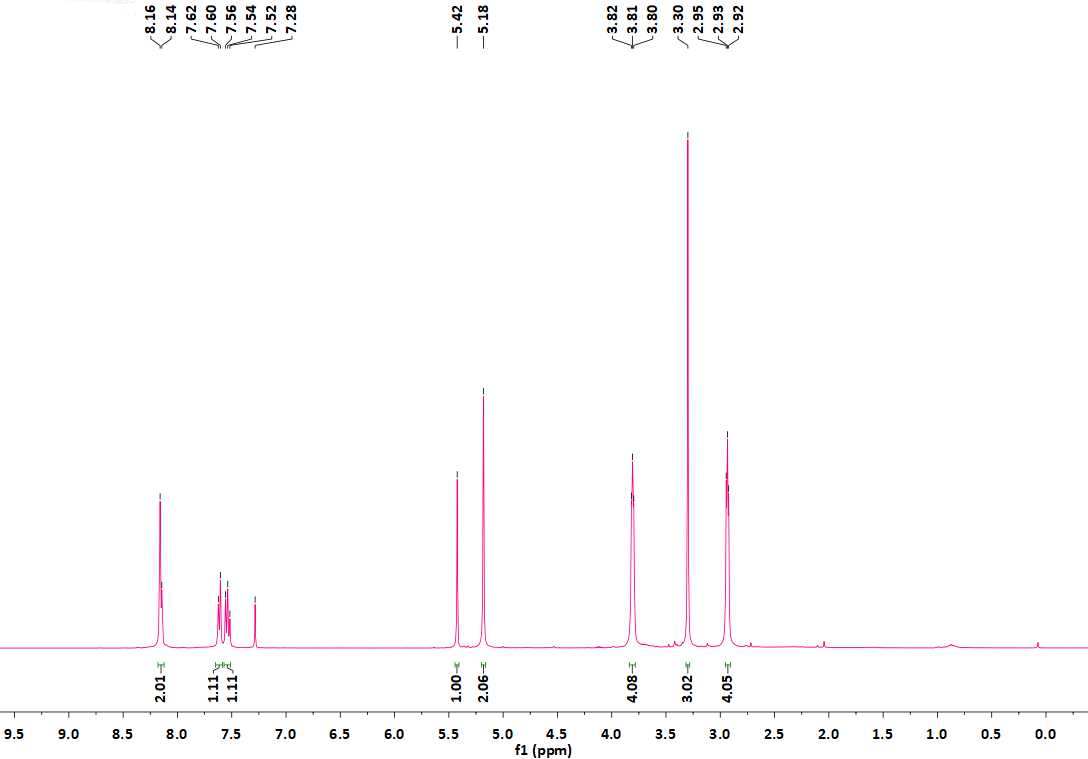


**Figure S37**: ^1^HNMR spectrum of **2h**


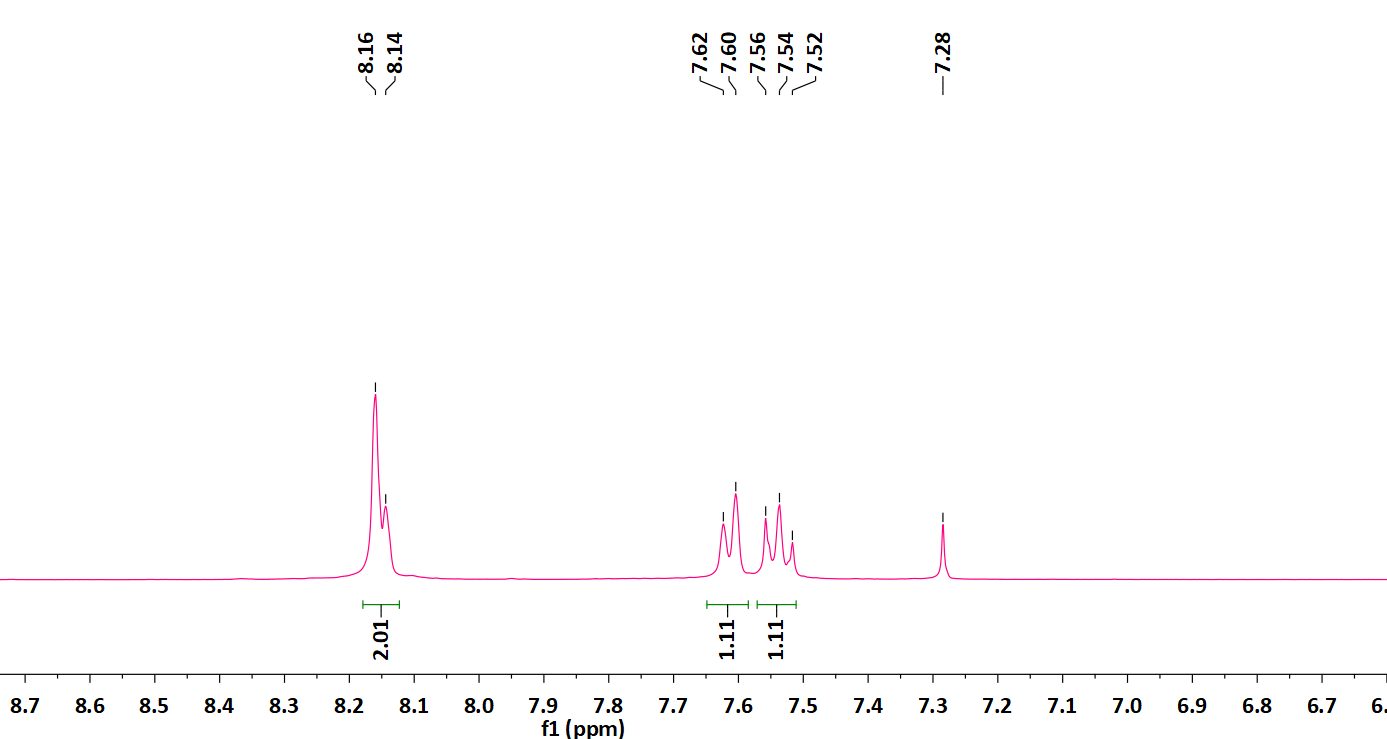


**Figure S38**: ^1^HNMR aromatic expand spectrum of **2h**


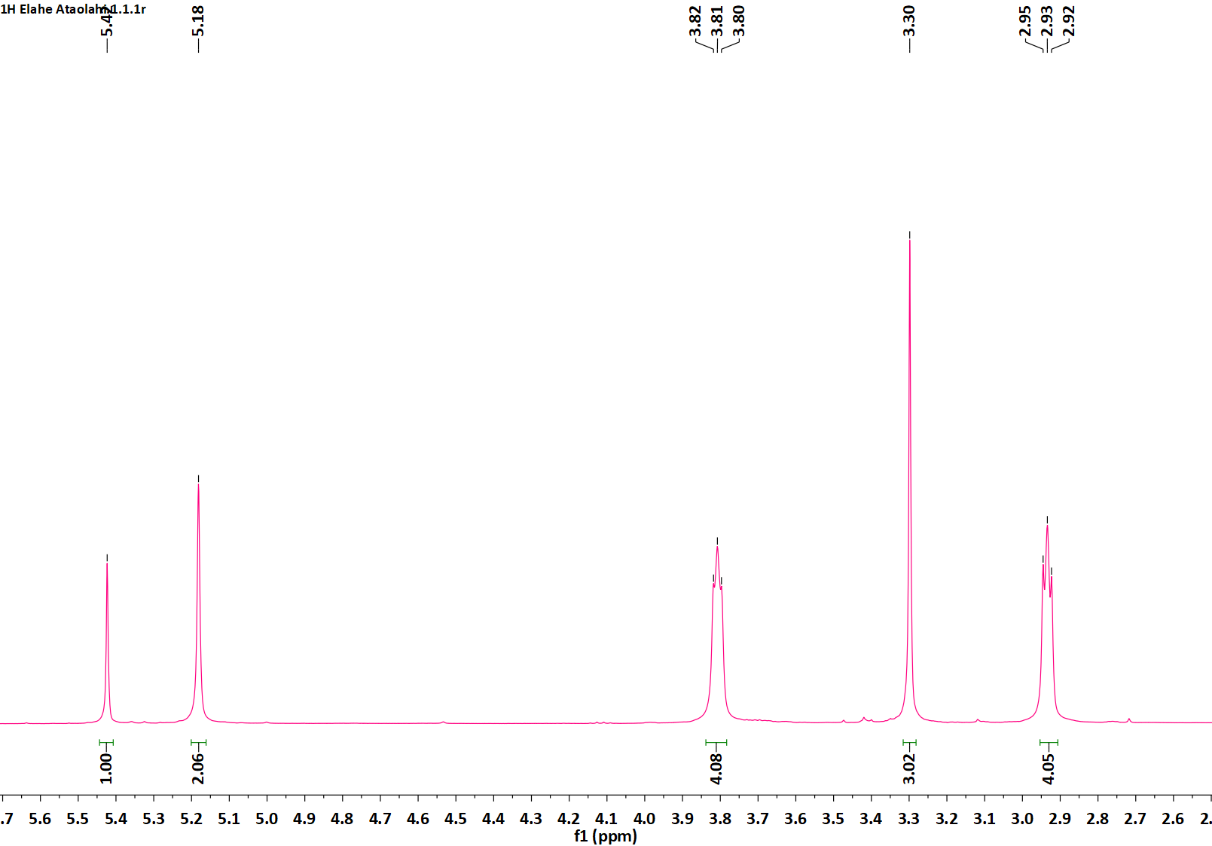


**Figure S39**: ^1^HNMR aliphatic expand spectrum of **2h**


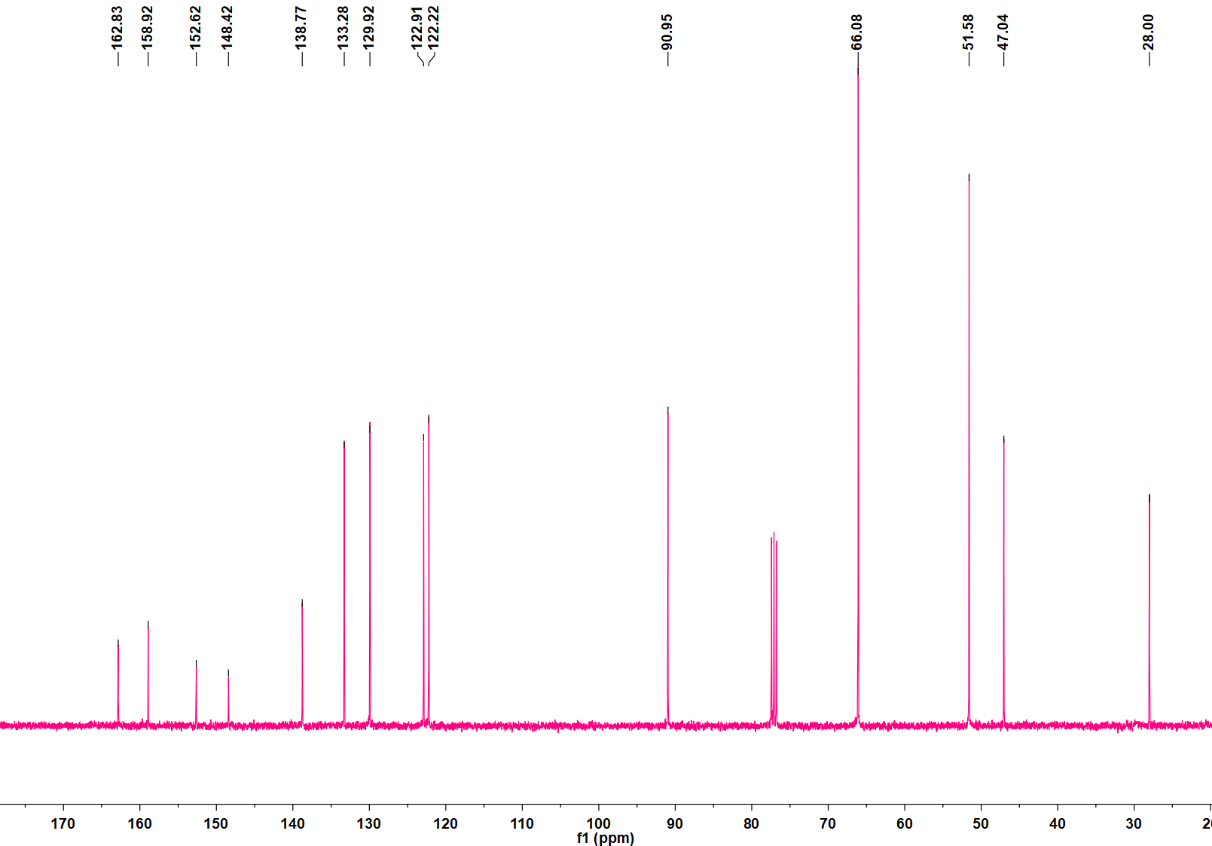


**Figure S40**: ^13^CNMR spectrum of **2h**


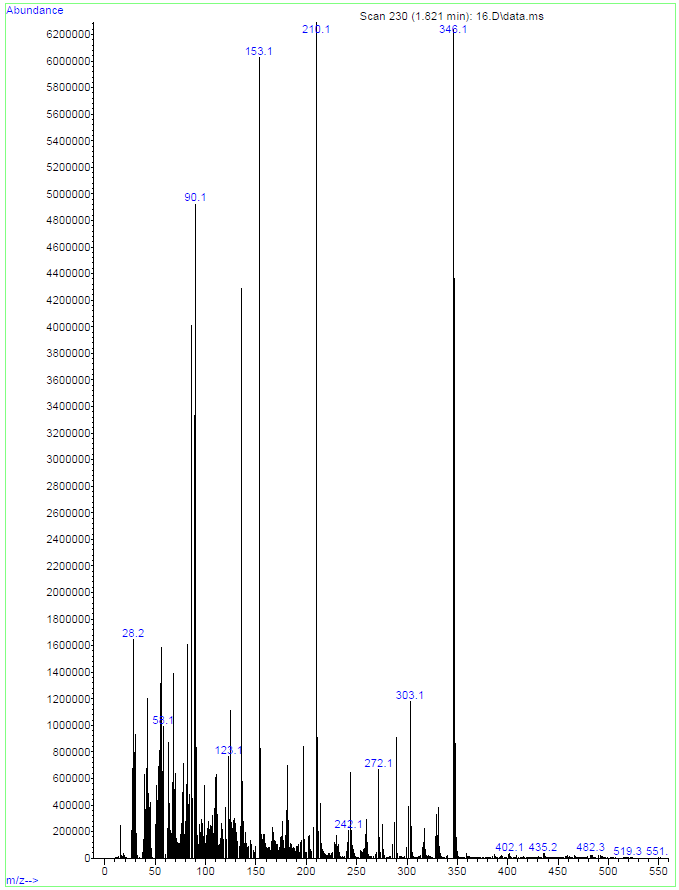


**Figure S41**: Mass spectroscopy of **2h**

1. *Corresponding authors:

   Soghra Khabnadideh, Tel: +98 -71-32424127-8; Fax: +98-71-32424126; E-mail: khabns@sums.ac.ir

   Zahra Rezaei, Tel: +98 -71-32424127-8; Fax: +98-71-32424126; E-mail: rezaeiza@sums.ac.ir

   a: These authors contributed equally to this work. [↑](#footnote-ref-1)
